# The Role of Inflammation in Cancer: Mechanisms of Tumor Initiation, Progression, and Metastasis

**DOI:** 10.3390/cells14070488

**Published:** 2025-03-25

**Authors:** Atsushi Nishida, Akira Andoh

**Affiliations:** Department of Medicine, Shiga University of Medical Science, Seta-Tsukinowa, Otsu 520-2192, Shiga, Japan; andoh@belle.shiga-med.ac.jp

**Keywords:** chronic inflammation, tumor microenvironment, cancer

## Abstract

Inflammation is an essential component of the immune response that protects the host against pathogens and facilitates tissue repair. Chronic inflammation is a critical factor in cancer development and progression. It affects every stage of tumor development, from initiation and promotion to invasion and metastasis. Tumors often create an inflammatory microenvironment that induces angiogenesis, immune suppression, and malignant growth. Immune cells within the tumor microenvironment interact actively with cancer cells, which drives progression through complex molecular mechanisms. Chronic inflammation is triggered by factors such as infections, obesity, and environmental toxins and is strongly linked to increased cancer risk. However, acute inflammatory responses can sometimes boost antitumor immunity; thus, inflammation presents both challenges and opportunities for therapeutic intervention. This review examines how inflammation contributes to tumor biology, emphasizing its dual role as a critical factor in tumorigenesis and as a potential therapeutic target.

## 1. Introduction

The link between inflammation and cancer has long fascinated researchers, tracing back to Rudolf Virchow’s 19th-century observation of leukocyte infiltration in tumor tissues [1]. Modern evidence unequivocally establishes inflammation as a pivotal factor in tumorigenesis, influencing every stage of cancer development, from initiation and promotion to malignant transformation, invasion, and metastasis. An inflammatory microenvironment is now recognized as a hallmark of nearly all cancers, even those lacking a direct causal link to chronic inflammation [2,3].

Environmental and lifestyle factors closely associated with inflammation contribute significantly to cancer incidence. Chronic infections, tobacco use, obesity, and dietary habits account for a large portion of the global cancer burden [4,5]. These factors sustain persistent inflammatory responses, fostering conditions that drive genetic mutations and tumor progression [6]. For instance, inflammatory bowel disease, chronic hepatitis, *Helicobacter*-induced gastritis, and *Schistosoma*-induced bladder inflammation substantially increase the risk of colorectal, liver, stomach, and bladder cancers, respectively. Conversely, some chronic inflammatory diseases, such as rheumatoid arthritis, exhibit weaker or even protective effects against cancer development [7].

Inflammation plays a dual role in cancer biology. While it can foster tumor growth by creating an environment rich in cytokines and growth factors that enhance cancer cell survival and proliferation, it can also activate antitumor immune responses, a mechanism harnessed in cancer immunotherapy [8]. This complexity underscores the need for a deeper understanding of the molecular and cellular mechanisms underlying inflammation’s impact on cancer [9].

Recent studies underscore the significant impact of inflammation on therapeutic outcomes. While acute inflammation can enhance immune responses to treatment, chronic inflammation may undermine therapeutic efficacy by fostering tumor recurrence and resistance. These insights suggest promising new strategies that target inflammation-driven pathways, opening novel avenues for cancer prevention and treatment [9].

## 2. Inflammation and Tumor Initiation: From DNA Damage to the Tumor Microenvironment

### 2.1. Inflammation and Genetic Instability

Inflammation plays a crucial role in the earliest stages of tumorigenesis by establishing conditions that promote genetic mutations and support the survival and proliferation of mutated cells. Tumor initiation occurs when a normal cell acquires genetic alterations that confer a selective growth and survival advantage over its neighboring cells. However, a single mutation is rarely sufficient to drive malignancy. Instead, most cancers arise from the accumulation of multiple genetic alterations, particularly in long-lived stem cells or transient amplifying cells that persist long enough to undergo successive genetic insults [10,11].

**Figure 1:**
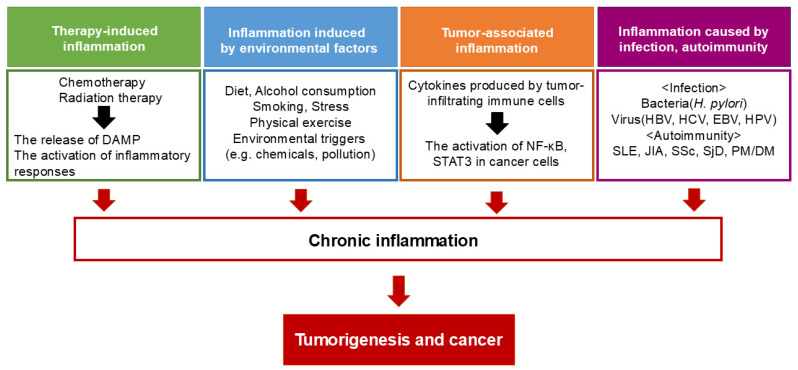
Inflammatory pathways in tumorigenesis and cancer. Chronic inflammation resulting from infections or autoimmune diseases often precedes tumorigenesis and contributes to its pathogenesis through mechanisms such as the induction of oncogenic mutations, genomic instability, early tumor promotion, and angiogenesis. Similarly, sustained exposure to environmental irritants or obesity can induce a state of low-grade chronic inflammation, which also predisposes individuals to tumorigenesis through these shared pathways. Tumor-associated inflammation is intrinsically linked to tumor progression, promoting neoangiogenesis, tumor growth, metastatic dissemination, localized immunosuppression, and exacerbated genomic instability. Furthermore, standard cancer treatments often trigger inflammatory responses due to treatment-induced trauma, necrosis, and tissue damage, which may contribute to tumor recurrence and therapeutic resistance. However, in specific contexts, treatment-induced inflammation can enhance antigen presentation and activate immune-mediated tumor eradication. DAMPs: danger-associated molecular patterns; SLE: systemic lupus erythematosus; JIA: juvenile idiopathic arthritis; SSc: systemic sclerosis; SjD: Sjögren’s disease; PM/DM: polymyositis/dermatomyositis; HBV: hepatitis B virus; HCV: hepatitis C virus; EBV: Epstein–Barr virus; HPV: human papillomavirus; *H. pylori*: *Helicobacter pylori*.

**Figure 2:**
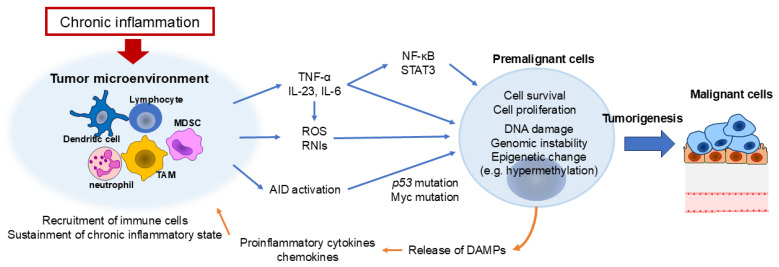
Chronic inflammation and tumor initiation. Inflammation plays a crucial role in all stages of tumorigenesis and contributes to tumor initiation by inducing mutations, genomic instability, and epigenetic modifications. Inflammatory responses activate tissue repair mechanisms, drive the proliferation of premalignant cells, and enhance tumor cell survival. Cytokines such as tumor necrosis factor-alpha (TNF-α), interleukin-6 (IL-6), and interleukin-23 (IL-23), along with the subsequent activation of nuclear factor kappa B (NF-kB) and signal transducer and activator of transcription 3 (STAT3), support tumor initiation. ROS: reactive oxygen species; RNI: reactive nitrogen intermediates; AID: activation-induced cytidine deaminase; DAMP: danger-associated molecular pattern; TAMs: tumor-associated macrophages; MDSCs: myeloid-derived suppressor cells.

Chronic inflammation, triggered by infections, irritants, or autoimmune diseases, actively contributes to tumorigenesis by creating a microenvironment conducive to genetic damage and aberrant cell proliferation (Figure 1) [9,12]. During inflammation, activated immune cells release reactive oxygen species (ROS) and reactive nitrogen intermediates, leading to DNA damage, genomic instability, and epigenetic modifications that elevate mutation rates [13,14]. In colitis-associated cancer, chronic inflammation is strongly linked to oxidative stress-induced mutations in tumor suppressor genes such as *p53*, as well as other cancer-related genes in intestinal epithelial cells (Figure 2) [15]. 

Beyond mutagenesis, inflammatory mediators influence epigenetic modifications that regulate gene expression. Tumor necrosis factor-alpha (TNF-α), a key proinflammatory cytokine, induces ROS production in epithelial cells, indirectly contributing to genetic instability. Additionally, cytokines such as interleukin (IL)-6 and IL-23 create a tumor-supportive niche by activating transcription factors like nuclear factor kappa-light-chain-enhancer of activated B cells (NF-κB) and signal transducer and activator of transcription 3 (STAT3). These factors regulate genes involved in cell survival, proliferation, and immune evasion, thereby fostering tumor initiation and progression [16,17].

Another crucial link between inflammation and tumor initiation is the disruption of DNA repair pathways. Persistent inflammatory stimuli can suppress mismatch repair enzymes while upregulating activation-induced cytidine deaminase (AID), an enzyme that promotes genomic instability. Under chronic inflammatory conditions, AID—normally restricted to antibody diversification in B cells—becomes aberrantly expressed in epithelial cells. This leads to mutations in key oncogenes and tumor suppressor genes, accelerating carcinogenesis [18,19].

### 2.2. Epigenetic Reprogramming and Tumor Promotion

Epigenetic modifications further drive inflammation-associated tumorigenesis by silencing tumor suppressor genes through DNA methylation or histone modification. For instance, murine models of chronic colitis show hypermethylation of promoter regions in tumor suppressor genes, an effect frequently mediated via NF-κB-dependent pathways. This epigenetic reprogramming underscores the long-term impact of chronic inflammation on genomic integrity and cancer susceptibility [20,21].

The effects of inflammation extend beyond mutagenesis, playing a pivotal role in the proliferation of mutated cell populations. Proinflammatory cytokines and growth factors secreted by immune and stromal cells enhance the proliferation of initiated cells, increasing the likelihood of additional mutations [22]. IL-6 and TNF-α drive epithelial cell proliferation and survival, facilitating the clonal expansion of premalignant cells. This process is further amplified by the recruitment of stromal and immune cells to inflamed tissues, where they secrete additional factors that nurture a tumor-promoting microenvironment [23,24]. Notably, inflammation can also endow epithelial cells with stem-like properties [25,26]. Cytokines such as IL-6 activate signaling pathways, including STAT3, to promote stem cell renewal and cellular reprogramming [27,28]. This expands the pool of cells vulnerable to oncogenic mutations, accelerating malignant progression. Additionally, inflammatory mediators enhance Wnt/β–catenin signaling, further driving stem-like phenotypes and uncontrolled proliferation [29].

### 2.3. Bidirectional Feedback Between Inflammation and DNA Damage

The relationship between inflammation and DNA damage is bidirectional. Tumor initiation itself generates inflammatory signals that exacerbate tissue damage and induce further mutations. For instance, DNA damage in hepatocytes exposed to carcinogens triggers necrotic cell death, leading to the release of damage-associated molecular patterns (DAMPs). These molecules activate immune cells, sustaining a proinflammatory response that fosters tumor growth [25,30]. Moreover, oncogenic mutations can directly initiate inflammatory signaling. Oncoproteins such as Ras and Myc upregulate the production of proinflammatory cytokines and chemokines, recruiting immune cells that perpetuate chronic inflammation. This creates a self-reinforcing cycle in which inflammation promotes genetic instability, while genetic alterations further sustain inflammation, driving tumor initiation and progression [31,32].

### 2.4. Role of Inflammation in Tumor Microenvironment and Metabolism

The critical role of the inflammatory microenvironment in early tumorigenesis is particularly evident in organ-specific cancers. In colitis-associated colorectal cancer, chronic inflammation precedes the formation of adenomas and carcinomas. Likewise, persistent liver inflammation caused by hepatitis B and C virus infections significantly increases the risk of hepatocellular carcinoma. These examples highlight the importance of deciphering the interactions between local inflammatory signals and tissue-specific factors that drive tumor initiation [33,34]. Enzymes derived from immune cells also contribute to tumor initiation. Proteases produced by macrophages and neutrophils, such as matrix metalloproteinases, facilitate tissue remodeling and compromise epithelial barriers, allowing inflammatory cells to infiltrate and increasing exposure to carcinogens. This disruption enhances the accumulation of mutagenic factors. Additionally, protease activity liberates growth factors from the extracellular matrix (ECM), further amplifying signaling pathways that promote epithelial proliferation and survival [35].

Beyond direct genetic and structural effects, inflammation also influences the metabolic state of epithelial cells during tumor initiation. Chronic inflammatory conditions often trigger metabolic reprogramming, characterized by increased glycolysis and lactate production [36]. These metabolic shifts not only fulfill the heightened energy demands of proliferating cells but also create an acidic microenvironment that fosters genetic instability and immune evasion [36,37]. Despite the well-established role of inflammation in promoting tumor initiation, its effects remain context-dependent. Acute inflammation, such as that induced by microbial components, can have protective effects by eliminating damaged cells and enhancing immune surveillance, thereby preventing tumor initiation [38]. Understanding the factors that dictate whether inflammation drives or suppresses tumorigenesis is essential for developing targeted therapeutic interventions.

## 3. Inflammation as a Catalyst for Tumor Growth and Progression

**Figure 3:**
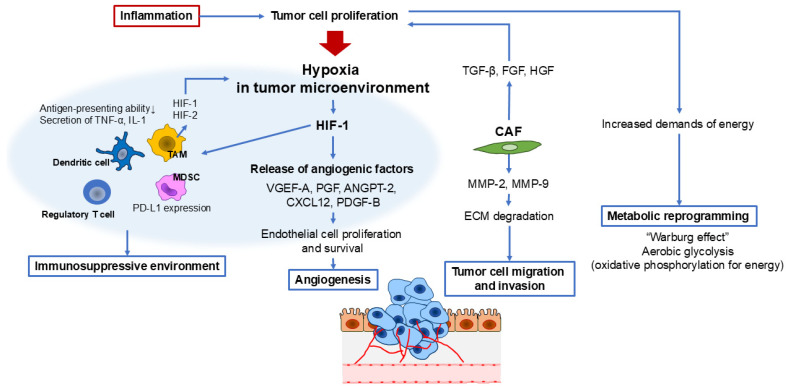
**Mechanism of tumor progression and invasion**. Inflammation promotes angiogenesis, suppresses local immune responses, and facilitates the development of a permissive tumor microenvironment that supports the persistence, expansion, and further genomic and epigenetic evolution of premalignant cells. Hypoxia within the tumor microenvironment induces the activation of hypoxia-inducible factor 1-alpha (HIF-1α), which promotes tumor angiogenesis and abnormal vascularization. Tumor cells secrete growth factors that stimulate proliferation and enhance invasive properties. Cancer-associated fibroblasts (CAFs) further contribute to extracellular matrix (ECM) remodeling by producing collagen and matrix metalloproteinases (MMPs). Metabolic reprogramming is essential for tumor growth, enabling cancer cells to meet increased energy, biosynthesis, and redox demands. Unlike normal cells, cancer cells predominantly rely on aerobic glycolysis, a phenomenon known as the Warburg effect. Hypoxia also creates an immunosuppressive tumor environment by recruiting myeloid-derived suppressor cells (MDSCs), tumor-associated macrophages (TAMs), and regulatory T cells (Tregs). Ultimately, chronic inflammation facilitates metastatic progression. Abbreviations: EMT: epithelial–mesenchymal transition; CTCs: circulating tumor cells; MMPs: matrix metalloproteinases; TGF-β: transforming growth factor-beta; FGF: fibroblast growth factor; HGF: hepatocyte growth factor; ECM: extracellular matrix; Treg: regulatory T cell; TAM: tumor-associated macrophage; MDSC: myeloid-derived suppressor cell.

Tumor promotion represents a crucial phase in cancer development, during which initiated cells progress into a fully developed primary tumor. This process is primarily driven by enhanced cell proliferation and reduced apoptosis, both of which are strongly influenced by inflammation (Figure 3) [38,39]. 

### 3.1. Angiogenesis in Tumor Growth

Inflammation-driven tumor promotion can also reactivate dormant premalignant lesions, stimulating their transition into an actively growing tumor [25]. The expansion of large tumors necessitates an increased blood supply, a demand primarily met through tumor-induced hypoxia. Hypoxia plays a pivotal role in tumor progression, significantly shaping the tumor microenvironment [40]. Most malignancies establish hypoxic conditions that suppress cell-mediated immunity and weaken overall immune responses [41,42]. Notably, hypoxia fosters immune tolerance by restricting immune cell infiltration and impairing their antitumor functions within tumors, further contributing to tumor progression [43].

HIFs serve as central regulators of angiogenesis by promoting the expression of proangiogenic factors such as vascular endothelial growth factor (VEGF), placenta growth factor (PGF), angiopoietin-2 (ANGPT-2), stromal-derived factor-1 (SDF-1, also known as CXCL-12), and platelet-derived growth factor-B (PDGF-B) [44,45,46]. These factors facilitate the angiogenic switch in tumors by binding to receptors on endothelial cells, pericytes, and vascular smooth muscle cells, thereby driving the formation of new blood vessels [44,45,46].

Among these mediators, VEGF-A is the most potent driver of hypoxia-induced angiogenesis [45,47]. The VEGF family—including VEGF-A, VEGF-B, VEGF-C, VEGF-D, and PGF—interacts with VEGF receptors (VEGFR-1 and VEGFR-2) on endothelial cells, activating signaling pathways that promote endothelial cell proliferation and survival. Specifically, VEGF-A stimulates the ERK and phosphoinositide 3-kinase (PI3K)/protein kinase B (AKT) pathways, enhancing endothelial cell proliferation and resistance to apoptosis (40). Additionally, VEGF-A regulates endothelial cell migration through Rho GTPases and facilitates ECM remodeling by inducing matrix metalloproteinases (MMP-2, MMP-9) and urokinase plasminogen activator [48,49].

Beyond its role in angiogenesis, VEGF-A also modulates vascular permeability, a critical process for maintaining tissue homeostasis [50]. However, in tumors, increased vascular permeability raises interstitial pressure, enabling tumor cell extravasation into the bloodstream and facilitating metastasis [51]. Unlike physiological angiogenesis, which generates functional vasculature, tumor-induced angiogenesis produces structurally and functionally abnormal blood vessels, leading to inefficient tumor perfusion and further contributing to tumor progression [44,52,53].

As tumors expand, they often develop hypoxic regions, raising the question of whether hypoxia itself directly drives tumor angiogenesis or whether it primarily initiates inflammatory signaling, which subsequently promotes angiogenesis. Studies indicate that inhibiting NF-κB or STAT3, neutralizing CCL2 or CXCL12, or depleting tumor-associated macrophages (TAMs) consistently impairs angiogenesis and suppresses tumor growth. These findings underscore the critical role of inflammatory mediators in regulating tumor-associated blood vessel formation and strongly suggest that inflammation plays a fundamental role in tumor angiogenesis [54,55].

### 3.2. Hypoxia and Immune Suppression in the Tumor Microenvironment

Hypoxia also profoundly influences the immune landscape of tumors by fostering an immunosuppressive microenvironment. Hypoxic tumor regions are frequently infiltrated by immunosuppressive cells, including myeloid-derived suppressor cells (MDSCs), TAMs, and regulatory T cells (Tregs) [56].

Hypoxia-inducible factor 1-alpha (HIF-1α) plays a key role in regulating MDSC function and differentiation within the hypoxic tumor microenvironment (TME). Evidence suggests that tumor-derived MDSCs exhibit greater immunosuppressive activity than their splenic counterparts, primarily due to HIF-1α-mediated upregulation of arginase activity and nitric oxide production. Additionally, hypoxia enhances PD-L1 expression in MDSCs, further promoting T-cell tolerance and immune evasion [57]. Notably, HIF-1α directly regulates PD-L1 expression by binding to hypoxia response elements in its promoter region, reinforcing its role in immune suppression within the TME [58].

Similarly, TAMs preferentially accumulate in hypoxic tumor regions, where they exhibit elevated expression of HIF-1 and HIF-2 [59]. These transcription factors play a pivotal role in enhancing the proangiogenic properties of macrophages, further driving tumor progression [60,61]. Additionally, hypoxia alters dendritic cell (DC) function by diminishing their antigen-presenting capacity while simultaneously increasing the secretion of proinflammatory cytokines, such as TNF and IL-1, as well as the inflammatory chemokine receptor CCR5 [62,63].

VEGF-A, which is highly abundant in hypoxic tumors, further suppresses DC maturation, impairing their ability to activate T cells and thereby facilitating immune evasion by tumor cells [62]. In the case of Tregs, hypoxia has been shown to upregulate Foxp3 expression through direct HIF-1α binding to the Foxp3 promoter, promoting both Treg differentiation and immunosuppressive function [64]. Moreover, hypoxia-induced upregulation of CCL28 in tumor cells enhances Treg recruitment, reinforcing the immunosuppressive tumor microenvironment [65].

Hypoxic stress has also been linked to the induction of the pluripotency factor NANOG in tumor cells, which subsequently activates transforming growth factor-beta (TGF-β1) expression and secretion. This mechanism, mediated by NANOG binding to the TGF-β1 promoter, contributes to immune suppression by reducing CD8^+^ T-cell infiltration while increasing the presence of TAMs and Tregs [66]. Experimental studies indicate that targeting this pathway can restore immune cell infiltration, reduce immunosuppressive cell populations, and enhance antitumor immunity in melanoma models [66].

### 3.3. Immune Modulation in the Tumor Microenvironment (TME)

The TME plays a pivotal role in tumor growth and progression, functioning as a dynamic ecosystem composed of cancer cells, immune cells, fibroblasts, endothelial cells, and the ECM. These components interact through intricate signaling networks that regulate cancer initiation, proliferation, invasion, and metastasis. While certain elements of the TME can exert tumor-suppressive effects, tumors often develop mechanisms to manipulate their microenvironment, fostering immune evasion, angiogenesis, and metastasis.

A key aspect of the TME is immune modulation. Initially, the immune system attempts to eliminate cancer cells through cytotoxic T cells and natural killer (NK) cells. However, tumors counteract these defenses by employing immune evasion strategies such as upregulating checkpoint molecules like PD-L1, recruiting immunosuppressive cells such as Tregs and MDSCs [67], and secreting anti-inflammatory cytokines. These adaptations establish an immunosuppressive environment that enables tumors to escape immune surveillance and sustain their growth. Cancer-associated fibroblasts (CAFs) are a major component of the TME and play a crucial role in tumor progression. Unlike normal fibroblasts, CAFs acquire an activated phenotype that supports cancer cell survival, invasion, and metastasis. They secrete growth factors such as TGF-β, fibroblast growth factor (FGF), and hepatocyte growth factor (HGF), which enhance tumor cell proliferation and invasive potential [22].

In addition to their role in tumor cell growth, CAFs contribute to ECM remodeling by producing collagen and MMPs, which degrade ECM barriers and facilitate cancer cell migration [68]. This remodeling process not only supports tumor expansion but also creates physical barriers that impede immune cell infiltration and the effective delivery of therapeutic agents.

Furthermore, CAFs modulate immune responses within the TME by secreting cytokines such as interleukin-6 (IL-6) and chemokines like CXCL12, which recruit immunosuppressive cells, including Tregs and MDSCs [22]. This contributes to the establishment of an immunosuppressive environment that further enhances tumor immune evasion.

Given their diverse protumorigenic functions, CAFs have emerged as a promising therapeutic target. Strategies aimed at inhibiting CAF activation, disrupting CAF-mediated signaling, or reprogramming CAFs into tumor-suppressive phenotypes are currently being explored to improve cancer treatment outcomes.

### 3.4. Metabolic Reprogramming and Therapy-Induced Inflammation

Metabolic reprogramming plays a fundamental role in tumor growth and progression, allowing cancer cells to meet increased demands for energy, biosynthesis, and redox balance. Unlike normal cells, which primarily rely on oxidative phosphorylation for energy production, cancer cells often shift toward aerobic glycolysis, a phenomenon known as the Warburg effect [69,70,71]. This metabolic adaptation enables tumors to sustain rapid proliferation even in oxygen-rich environments. Additionally, cancer cells exploit alternative metabolic pathways, including glutamine metabolism and lipid biosynthesis, to support their growth and survival.

As tumors progress, their metabolic profiles become increasingly heterogeneous, shaped by genetic mutations, microenvironmental conditions, and selective pressures [72]. Early-stage tumors primarily rely on glucose and amino acid metabolism to fuel biosynthetic pathways. However, as tumors invade surrounding tissues and metastasize, they frequently develop dependencies on oxidative phosphorylation and lipid metabolism to withstand oxidative stress and adapt to new environments [73]. This metabolic plasticity poses challenges for therapeutic interventions, as distinct tumor subtypes—and even different regions within the same tumor—may exhibit unique metabolic vulnerabilities.

A key aspect of metabolic reprogramming is its interaction with the tumor microenvironment. Hypoxia, a hallmark of solid tumors, stabilizes HIF-1α, which subsequently upregulates glycolysis- and angiogenesis-related genes [74]. Additionally, stromal and immune cells within the tumor microenvironment contribute to metabolic alterations by supplying nutrients or modulating metabolic pathways. For instance, TAMs secrete cytokines that promote lipid metabolism and redox homeostasis, thereby enhancing tumor resilience [75].

The dual role of inflammation in tumorigenesis is evident in its ability to sustain chronic inflammatory responses while also modulating immune surveillance. Although inflammation is often linked to tumor-promoting effects, it can also stimulate antitumor immunity under specific conditions [9,33]. Certain cytokines produced during inflammation enhance the activity of cytotoxic T cells and natural killer cells, counteracting tumor-promoting mechanisms [76,77]. However, the overall impact of inflammation on tumor progression is highly context-dependent, influenced by tumor type, developmental stage, and the composition of the tumor microenvironment [77]. This complexity underscores the need for targeted therapeutic strategies that selectively disrupt protumorigenic inflammatory pathways while preserving or enhancing antitumor immune responses.

Therapy-induced inflammation plays a significant role in tumor promotion. Cancer treatments such as chemotherapy and radiation cause substantial tissue damage, triggering the release of DAMPs and activating inflammatory responses [78,79]. While these responses can enhance tumor antigen presentation and stimulate antitumor immunity under certain conditions, they may also paradoxically support tumor survival and therapy resistance by activating prosurvival signaling pathways [21].

### 3.5. Tumor Communication and Invasion Mechanisms

Intracellular communication between cancer cells and their microenvironment is essential for tumor progression [80,81]. Tunneling nanotubes (TNTs) and tumor microtubes (TMs) play a critical role in this process by facilitating intercellular communication and enhancing cancer cell invasion. These dynamic membrane structures enable the transfer of cellular components—including organelles, proteins, and signaling molecules—thereby promoting tumor cell survival, adaptation, and coordination.

TMs, in particular, share structural similarities with neuronal growth cones, which are essential for neuronal migration during development [82]. Studies have shown that glioma cells utilize TMs as conduits for invasion, with these microtubules guiding the movement of cell nuclei during division. The interconnected network formed by TMs allows glioma cells to function as a synchronized unit, facilitating tumor expansion. Notably, the extent of TM formation correlates with tumor malignancy, as cells within these networks exhibit heightened invasive potential [82].

Glioma cells exhibit a preference for perivascular spaces and neuronal axons as pathways for invasion [83]. The perivascular niche serves as a reservoir for cancer stem cells, contributing to long-term tumor persistence and therapeutic resistance. Additionally, TMs facilitate long-range calcium signaling within astrocytoma cells, further supporting tumor survival and adaptation within the brain microenvironment [83].

Several molecular regulators play key roles in TM formation and function. Cx43 gap junctions stabilize TMs, enhancing intercellular communication and coordination among glioma cells [83]. The membrane protein tweety-homolog 1 (Ttyh1), which is involved in neuronal development, is critical for TM-dependent invasion and proliferation [84]. Furthermore, growth-associated protein 43 (GAP-43) is highly expressed in glioma cells, promoting TM formation and facilitating cell migration. Inhibition of these molecules has been shown to reduce tumor progression, highlighting their significance in tumor biology [85].

Similarly, TNTs have been implicated in mesothelioma cell invasion [86]. Time-lapse imaging studies reveal that TNTs form at the invasive tumor front, assisting in cell migration and tumor expansion [86]. These findings emphasize the critical role of TNTs and TMs as key mediators of cancer progression, enabling cellular interactions that enhance malignancy and therapy resistance.

## 4. Inflammation as a Key Driver of Cancer Metastasis

**Figure 4:**
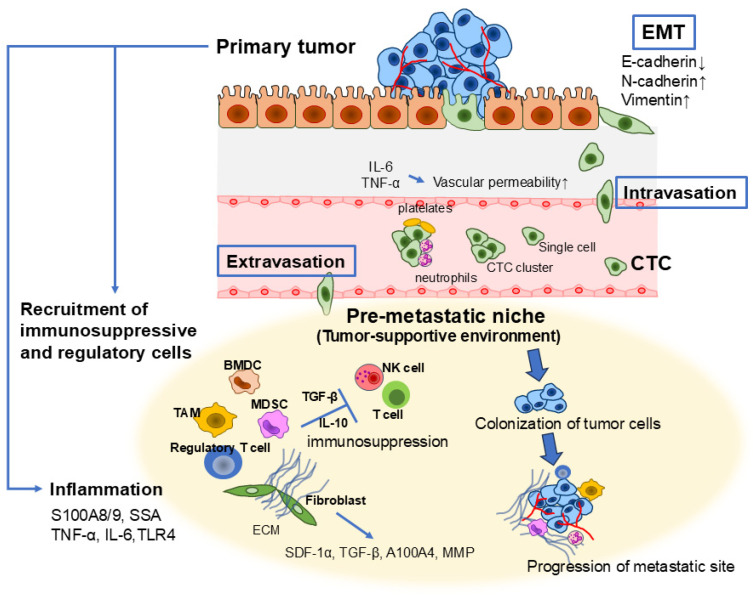
**Mechanism of tumor metastasis.** Epithelial–mesenchymal transition (EMT) is a critical process in tumor metastasis, conferring cancer cells with motility, invasiveness, and resistance to cellular stress. During EMT, epithelial cells lose polarity and adhesion, adopting a mesenchymal phenotype with increased mobility. This transition involves the downregulation of E-cadherin and the upregulation of mesenchymal markers, facilitating tumor cell dissemination. Circulating tumor cells (CTCs) detach from the primary tumor and enter the bloodstream or lymphatic system. Their survival is enhanced through clustering or interactions with platelets, while neutrophils contribute to cluster formation and suppress leukocyte activation. Additionally, primary tumors can modify distant organ microenvironments, forming a premetastatic niche (PMN) that facilitates future colonization. CTC: circulating tumor cell; PMN: premetastatic niche.

Metastasis, the dissemination of cancer cells from the primary tumor to distant organs, is the leading cause of cancer-related mortality, accounting for over 90% of cancer deaths [87,88]. Chronic inflammation plays a pivotal role in metastasis by orchestrating intricate interactions between immune regulation, cancer progression, and inflammatory signaling pathways. Dysregulated activation of TLR4 signaling in tumor cells can initiate inflammatory responses that enhance resistance to cell death while promoting proliferation and invasion. Similarly, aberrant activation of TLR4 and other innate immune sensors in immune cells contributes to chronic inflammation, further accelerating tumor progression and metastasis [89]. Recent studies highlight the critical role of interactions among cancer cells, immune and inflammatory cells, and stromal components within the tumor microenvironment in driving metastatic progression [22,39]. Metastasis is a complex, multistep process comprising several key phases: EMT, intravasation into the bloodstream or lymphatic system, survival in circulation, extravasation into distant tissues, and eventual formation of metastatic niches (Figure 4) [90,91].

### 4.1. Epithelial–Mesenchymal Transition (EMT) in Tumor Metastasis

EMT is a fundamental process in tumor metastasis, equipping cancer cells with increased motility, invasiveness, and resistance to external stressors. During EMT, epithelial cells lose their polarity and intercellular adhesion properties, transitioning into a mesenchymal phenotype characterized by enhanced mobility and invasiveness [90,92]. This transformation is governed by a complex network of transcription factors, signaling pathways, and microenvironmental cues that collectively facilitate tumor progression. As a developmental process, EMT reduces cell–cell adhesion and enhances cellular plasticity, allowing cancer cells to navigate through tissue barriers and spread to distant sites.

One of the critical functions of EMT in metastasis is facilitating tumor cell dissemination. As cancer cells undergo EMT, they detach from the primary tumor by downregulating adhesion molecules such as E-cadherin while upregulating mesenchymal markers, including N-cadherin, vimentin, and fibronectin, along with cell-associated MMP activity. Morphologically, this transition leads to the loss of the characteristic polygonal, cobblestone-like epithelial structure, giving rise to elongated, fibroblast-like cells with enhanced migratory and invasive capabilities [93]. Furthermore, EMT endows cancer cells with resistance to apoptosis and cellular senescence, further supporting tumor survival and progression [94]. The shift in gene expression that suppresses the epithelial phenotype while promoting mesenchymal characteristics is regulated by key transcription factors, such as SNAIL, TWIST, and zinc-finger E-box-binding (ZEB) proteins. These factors are activated early in the EMT process and play essential roles in development, fibrosis, and cancer. Their expression patterns vary across different cell and tissue types, and their specific contributions to EMT depend on the signaling pathways that drive the transition [95].

Emerging evidence highlights a strong connection between cancer-related EMT and chronic inflammation. Various inflammatory factors—including soluble mediators, oxidative stress, and hypoxia—have been shown to promote EMT-like characteristics in cancer cells [96]. In turn, EMT-transformed cells can secrete elevated levels of proinflammatory molecules such as cytokines, chemokines, and MMPs, thereby sustaining a persistent inflammatory state that fuels tumor progression. Numerous studies have established a direct link between EMT and inflammation-related soluble mediators. TNF-α, particularly in combination with TGF-β or other inflammatory signals, has been shown to drive EMT [97]. For instance, a combination of TGF-β, interferon-alpha (IFN-α), and TNF-α has been used to successfully induce EMT-like changes in human cancer cell lines in vitro [98].

TNF-α-driven EMT has been observed across various cancer cell lines, including hepatocellular carcinoma [99], breast cancer [100], lung cancer [101], and thyroid cancer, where it functions in conjunction with interferon-gamma (IFN-γ) [102]. Notably, in renal cell carcinoma models, TNF-α-induced EMT signaling requires the expression of CXCR2, CXCR3, and their respective ligands, underscoring the critical role of chemokine interactions in facilitating this transition [103]. Several studies have established a causal relationship between the IL-1β/IL-1R signaling pathway and the induction of EMT-like characteristics in (pre)cancerous cells in vitro [104]. Furthermore, IL-6 signaling and EMT exhibit a reciprocal relationship in cancer. IL-6 promotes EMT through the JAK2/STAT3 pathway, driving mesenchymal traits in various cancers [105,106]. Conversely, EMT enhances IL-6 expression via transcription factors such as TWIST [105,107], creating a feedback loop that sustains tumor progression through STAT, ERK, and AKT pathways [108]. Th17 cell infiltration within tumors is associated with elevated levels of IL-23, IL-17, IL-1β, and IL-6. In esophageal cancer, IL-23 expression has been linked to the presence of metastases [109]. Mechanistically, IL-23 promotes EMT-like changes via activation of the Wnt/β-catenin signaling pathway [109]. Similarly, IL-17 enhances EMT in cancer cell lines and gastric cancer stem cells, increasing their invasiveness and activating STAT3 signaling [110,111]. These findings underscore the pivotal role of proinflammatory cytokines in driving EMT and tumor progression. TNF-α, IL-1β, IL-6, and Th17-associated cytokines contribute to EMT through distinct yet interconnected signaling pathways, fostering an inflammatory tumor microenvironment that enhances invasiveness, metastasis, and resistance to therapy.

### 4.2. Intravasation and Circulation of Cancer Cells

Once cancer cells gain mobility across the ECM, they intravasate into blood vessels or lymphatics. Inflammation plays a significant role in this process by increasing vascular permeability, thereby facilitating cancer cell invasion into blood vessels [39,112]. Proinflammatory cytokines, including TNF-α and IL-6, disrupt endothelial cell junctions, making it easier for cancer cells to enter circulation [113,114]. Integrins, essential adhesion receptors, are pivotal in cancer cell intravasation and progression, from primary tumor growth to metastasis [115]. Abnormal integrin expression is frequently observed in tumors, where these receptors enhance oncogenic growth factor receptor (GFR) signaling, as well as GFR-dependent cell migration and invasion [115]. Additionally, integrins play a crucial role in the colonization of metastatic sites and support the survival of circulating tumor cells (CTCs) in an anchorage-independent manner. At metastatic sites, cancer cells utilize E-cadherin to detach, spread, and establish new growths [116]. This process enhances metastatic cell survival and protects against apoptosis induced by reactive oxygen species. As a result, targeting E-cadherin in metastatic breast cancer cells may represent a promising therapeutic approach [116].

CTCs are cancer cells that detach from the primary tumor and enter the bloodstream or lymphatic system. While traveling through the bloodstream, CTCs encounter significant challenges, including immune surveillance, shear stress, and anoikis (detachment-induced cell death). Most CTCs do not survive in circulation, with only a small fraction persisting and successfully invading distant organs. One key survival strategy employed by CTCs is immune evasion. EMT-associated tumor cells often downregulate epithelial markers such as E-cadherin while upregulating mesenchymal markers like vimentin, making them less detectable by immune cells [117,118]. Moreover, EMT promotes the secretion of immunosuppressive cytokines, which inhibit cytotoxic T-cell responses.

Another key survival mechanism involves the formation of CTC clusters or interactions with platelets. While most CTCs travel through the bloodstream as single cells, some move in clusters [119]. These clustered CTCs have a significantly higher potential to establish metastases. In addition to cancer cells, these clusters contain stromal cells and immune components from their original microenvironment, contributing to their heterogeneity and enhancing their survival [119,120]. Neutrophils also play a role in cluster formation by suppressing leukocyte activation, further supporting CTC survival [121].

Platelets contribute to CTC survival as well as their seeding and outgrowth at secondary sites [122,123]. By forming protective aggregates around CTCs, platelets shield them from immune surveillance, particularly from natural killer cells. CTCs facilitate this aggregation by releasing prothrombotic and procoagulant microparticles or by expressing tissue factor [124]. Additionally, platelet-derived factors such as TGF-β have been shown to accelerate EMT in CTCs, enhancing their invasion and metastatic potential [125]. Furthermore, platelets are believed to protect CTCs from mechanical stress and promote resistance to anoikis, a process linked to the activation of the YAP1 pathway [126]. Some CTCs also enter a dormant state, allowing them to evade immune detection and later reactivate to form metastatic colonies. These survival adaptations make CTCs highly effective metastatic seeds, capable of enduring circulation and colonizing distant organs. Understanding these mechanisms provides crucial insights into tumor metastasis and potential strategies for disrupting CTC survival [117,118].

### 4.3. Extravasation of Cancer Cells and the Role of the Premetastatic Niche

After surviving in circulation, CTCs become lodged in the small capillaries of distant organs, where they can extravasate and initiate secondary tumorigenesis. This process is strongly influenced by the local microenvironment, which plays a key role in determining whether tumor cell colonization occurs. Primary tumors can modify the microenvironment of distant organs, creating favorable conditions for tumor cell colonization—known as the premetastatic niche (PMN)—even before tumor cells reach these sites [127].

The PMN is a dynamic and multifaceted microenvironment shaped by coordinated interactions among various bone marrow-derived cells (BMDCs) and multiple immune cell types. Cells within the premetastatic niche actively remodel the local microenvironment by releasing inflammatory cytokines, growth factors, and proangiogenic factors. This process fosters tumor cell colonization, supports their proliferation, and ultimately accelerates metastatic progression [128]. Chemokines and cytokines secreted by tumor cells promote the recruitment of MDSCs, TAMs, Tregs, and tumor-associated neutrophils to distant secondary sites. These immunosuppressive and regulatory cell populations contribute to PMN formation, thereby creating a microenvironment conducive to metastatic progression [129,130]. Furthermore, fibroblasts within the premetastatic niche play a crucial role in shaping a tumor-supportive microenvironment by secreting inflammatory cytokines and growth factors, including stromal cell-derived factor-1α (SDF-1α), TGF-β, and S100A4. Additionally, they contribute to ECM remodeling by expressing fibronectin and MMPs, thereby facilitating tumor cell invasion and metastasis [131].

The inflammatory PMN plays a crucial role in promoting metastasis. Proinflammatory mediators such as S100A8/A9, secreted within lung premetastatic niches, have been shown to induce the expression of serum amyloid A (SAA)3, which subsequently facilitates the recruitment of Mac1^+^ myeloid cells to these sites via TLR4 signaling [132]. These recruited myeloid cells contribute to premetastatic niche formation under inflammatory conditions, thereby enhancing the migration of primary tumor cells to secondary lung sites [132]. Additionally, TNF-α-driven S100A8-SAA3-TLR4 signaling has been found to activate Clara cells, a non-immune cell population, leading to sustained SAA3 expression and the perpetuation of an inflammatory microenvironment that supports lung metastasis [133]. Furthermore, S100A4 can induce SAA1 and SAA3 expression via TLR4 and NF-κB signaling in an organ-specific manner, suggesting a potential link between inflammation and tumor metastasis in premetastatic niches, which has been associated with poor prognosis in human colon carcinomas [134]. The accumulation and activation of neutrophils within the premetastatic niche play a significant role in facilitating tumor metastasis. Neutrophil-derived inflammatory responses help shape a microenvironment conducive to metastatic progression. Specifically, leukotrienes secreted by neutrophils have been implicated in promoting the colonization and metastatic spread of breast cancer cells in the lungs [135]. CD11b^+^Gr-1^+^ cells within the lung premetastatic niche further contribute to the establishment of an inflammatory and proliferative microenvironment by upregulating MMPs and Th2 cytokines while simultaneously suppressing IFN-γ production, thereby facilitating tumor progression [136]. Additionally, increased IL-6 expression, driven by TLR5 signaling and hypoxic conditions within the premetastatic niche, has been identified as a key factor in promoting tumor-associated inflammation [137].

### 4.4. Immune Modulation and Hypoxia in the Metastatic Niche

The ECM within the metastatic niche undergoes extensive remodeling to create a favorable environment for metastatic colonization [77]. MMPs degrade ECM components, facilitating tumor cell invasion and altering the biochemical properties of the niche. Additionally, stromal fibroblasts contribute to ECM reorganization by producing cytokines and growth factors that promote tumor cell proliferation and survival.

Another critical aspect of the metastatic niche is immune modulation. Immune cells, including MDSCs, Tregs, and TAMs, accumulate within the niche and suppress antitumor immune responses. These cells secrete immunosuppressive cytokines such as TGF-β and IL-10, which inhibit cytotoxic T-cell activity and promote immune evasion [138].

Hypoxia within the metastatic niche further accelerates tumor progression. Low oxygen levels stabilize HIF-1α, which upregulates genes involved in angiogenesis, metabolic adaptation, and apoptosis resistance. This hypoxic environment supports metastatic cell survival and prepares the niche for sustained tumor growth.

The role of the immune system in metastasis is dual-faceted. On one hand, inflammation promotes metastasis by supporting cancer cell survival, migration, and proliferation. On the other, the immune system provides protective surveillance [77]. In some cases, infiltrating T cells, particularly cytotoxic CD8^+^ T cells, can recognize and eliminate metastatic cancer cells [139]. However, as tumors progress, they often develop mechanisms to evade immune surveillance, such as inducing immune suppression via Tregs and secreting immunosuppressive cytokines like TGF-β [9,140].

Understanding the complex relationship between inflammation and metastasis provides key insights into potential therapeutic strategies. Targeting proinflammatory pathways involved in metastasis, such as inhibiting cytokines and immune cells that promote tumor spread, may help modulate metastatic progression [9,114]. Additionally, therapies that enhance immune surveillance, including checkpoint inhibitors, could counteract the immune evasion mechanisms employed by metastatic cancer cells [141].

## 5. Cytokine Signaling in Tumor Promotion and Inflammation

### 5.1. Role of Cytokines in Tumor Promotion

Protumorigenic cytokine signaling plays a critical role in cancer progression by driving inflammatory processes that sustain tumor growth, angiogenesis, and metastasis. Cytokines, signaling molecules released by immune and inflammatory cells, are essential in the complex interactions within the tumor microenvironment [114]. These molecules activate various transcription factors, including NF-κB, STAT3, and AP-1, which regulate genes involved in cell survival, proliferation, and tumor progression. Through these signaling networks, cytokines orchestrate multiple tumor-promoting processes, ultimately enhancing the malignant potential of tumors [114].

### 5.2. Key Cytokines and Their Mechanisms

A key aspect of tumor-promoting cytokine signaling is the activation of transcription factors by inflammatory cytokines such as TNF-α, IL-6, IL-1β, and IL-23. These cytokines are produced by tumor-associated immune cells, including macrophages and T cells recruited to the tumor site in response to signals from the tumor and surrounding stromal cells [114,142]. Early evidence linking inflammation to tumor promotion came from studies on TNF-α’s role in the initiation and progression of skin cancer in murine models. TNF-α primarily promotes tumorigenesis by activating AP-1 and NF-κB. Although NF-κB can suppress tumorigenesis in certain contexts, its activation within the tumor microenvironment often establishes a proinflammatory milieu that supports tumor growth and progression [143].

*NFKB1* and *STAT3* are classified as non-classical oncogenes because their activation typically results not from direct genetic mutations but from signals within the tumor microenvironment, including those from immune and tumor cells. These transcription factors regulate genes essential for inflammation, immune evasion, survival, and metastasis [144,145]. For example, STAT3 activation upregulates the production of IL-6 and other cytokines that promote tumor cell proliferation and angiogenesis. In turn, these cytokines recruit additional immune cells, perpetuating a cycle of chronic inflammation that drives tumor growth [146].

Another key cytokine involved in tumor promotion is IL-23, which is predominantly produced by TAMs. IL-23 plays a significant role in the tumor microenvironment by stimulating the production of other inflammatory cytokines, such as IL-17, to further tumor progression. In animal models of skin carcinogenesis, IL-23 has been shown to increase tumor multiplicity and growth. Its tumor-promoting effects are likely mediated by the activation of T helper 17 (Th17) cells, which secrete cytokines that support tumor growth. IL-23 also influences other immune cells, including cytotoxic T lymphocytes and myeloid cells, sustaining the inflammatory environment within the tumor [147].

### 5.3. Immunosuppression and Therapeutic Implications

The relationship between cytokine signaling and tumor promotion is further complicated by the presence of MDSCs, immune cells that inhibit antitumor immunity. MDSCs secrete various factors, such as arginase-1 and indoleamine 2,3-dioxygenase, which suppress T-cell activation and create a tumor-supportive immune environment [9,148]. This immunosuppressive activity facilitates immune evasion, allowing malignant cells to bypass immune mechanisms that would otherwise recognize and eliminate them. Additionally, cytokine production by tumor cells themselves is a key mechanism of tumor promotion. While immune cells are the primary source of proinflammatory cytokines, many cancer cells, particularly in advanced stages, produce their own cytokines to sustain survival and proliferation. For example, IL-6 is produced by various tumor types, including HCC and colorectal cancer, where it exerts an autocrine function to promote cancer cell survival and resistance to apoptosis [33,149]. This cytokine production enables tumors to evade the host immune response and persist in a hostile microenvironment.

Cytokines also play a crucial role in angiogenesis. Inflammation-driven angiogenesis is essential for rapid tumor growth and metastasis. Tumor cells, immune cells, and stromal cells collectively contribute to the release of proangiogenic factors, such as vascular endothelial growth factor, which stimulates new blood vessel formation. The NF-κB and STAT3 signaling pathways regulate the expression of these proangiogenic factors, thereby enhancing the tumor blood supply and supporting metastasis [45,150].

A key feature of inflammation-driven tumor promotion is the ability of tumor-promoting cytokines to establish a self-perpetuating feedback loop. As tumors grow and invade surrounding tissues, they release signals that recruit additional immune cells, which, in turn, secrete cytokines that further support tumor growth. This positive feedback loop can lead to chronic inflammation, which not only promotes tumor progression but also reduces the efficacy of anticancer therapies [9]. Treatments that induce tumor cell death, such as chemotherapy and radiation, can trigger an inflammatory response that enhances tumor survival and resistance to subsequent treatment [77]. This resistance is partly driven by the activation of prosurvival signaling pathways, including those mediated by NF-κB and STAT3, which enable residual cancer cells to persist and proliferate after therapy.

The complex interplay between cytokine signaling, inflammation, and tumor promotion underscores the importance of targeting these pathways for therapeutic intervention. Several strategies aimed at blocking the signaling pathways driven by protumorigenic cytokines are currently being explored. Preclinical studies on NF-κB and STAT3 inhibition have demonstrated the potential of this approach in suppressing tumor growth and reducing inflammation. However, these strategies must be carefully balanced, as prolonged inhibition of these pathways can lead to immune deficiencies and other adverse effects, such as neutrophilia and exacerbated acute inflammation [149]. Therapies that block the recruitment and activation of inflammatory cells within the tumor microenvironment are also under investigation. For instance, inhibiting chemokine signaling to prevent myeloid cell recruitment may reduce tumor-associated inflammation and limit tumor progression [148]. Additionally, anticytokine therapies, such as antibodies that neutralize TNF-α, IL-6, and other proinflammatory cytokines, are already in clinical use for treating chronic inflammatory diseases and are being evaluated for their potential to suppress tumor growth and metastasis in various cancers [27].

## 6. Other Factors Associated with Cancer Progression

### 6.1. Activin A

Activin A, a multifunctional cytokine of the TGF-β superfamily, plays a crucial role in cell proliferation, differentiation, apoptosis, and immune regulation. Initially identified for its role in reproductive physiology, it is now recognized as a key modulator in embryonic development, tissue repair, and inflammation. In cancer biology, Activin A influences tumor progression by affecting cell migration, invasion, and the tumor microenvironment.

The role of Activin A in the cell growth regulation is complex and context-dependent. It can induce apoptosis and inhibit proliferation in some cells, including normal hepatocytes and certain cancer cell lines, such as prostate, breast, and colon tumors, as well as diffuse large B-cell lymphoma [22,51,52,54,91]. Conversely, it enhances proliferation in lung fibroblasts and certain lung cancer cells. The mechanisms underlying these opposing effects remain unclear, highlighting the need for further research into their regulatory pathways.

Activin A’s impact on tumor progression varies across cancer types. In some epithelial tumors, it exerts protective effects by inducing cell cycle arrest and inhibiting proliferation [22,51,52,54,91]. For example, it suppresses growth in patient-derived prostate cancer cells and non-invasive LNCaP cells [45,93,94,95], whereas aggressive PC3 prostate cancer cells exhibit increased proliferation upon Activin A exposure [69,98]. Endoglin, a TGF-β co-receptor, modulates Activin A signaling, with ActRIIA co-expression suppressing invasion in prostate cancer cells, while interaction with ActRIIB does not [99,100].

Conversely, Activin A promotes invasion and metastasis in certain cancers. In lung adenocarcinoma and oral squamous cell carcinoma, its overexpression correlates with lymph node involvement and poor prognosis [9,11,13,15,85,104,105]. Similarly, in head and neck squamous cell carcinoma, high Activin A levels are associated with reduced survival rates [9,26,27,28,85,106]. Functional studies show that recombinant Activin A enhances proliferation in lung cancer cell lines (H460, SKLU1) [26,29,30,63,86,107,108,109] and promotes invasion in ovarian cancer cell lines (SKOV-3, OCC1) [110].

Activin A’s invasive properties are primarily mediated through MMP activity. It induces MMP-7 expression via c-Jun/Smad signaling, facilitating ECM degradation and enhancing invasion [31,32,33,34,35,87,88,89,109]. Additionally, it upregulates N-cadherin, a mesenchymal marker linked to invasiveness, independent of E-cadherin expression [44,45,46,47,50,91,112].

During tumor progression, cancer cells may develop resistance to Activin A’s growth-inhibitory effects by downregulating ALK4 or upregulating inhibitors such as follistatin [58,59,102,121]. These adaptive mechanisms allow tumors to evade Activin A signaling, highlighting its complex and context-dependent role in cancer development. Understanding these regulatory pathways may offer new insights for therapeutic strategies targeting Activin A across different cancer types.

### 6.2. NRF2

Nrf2 plays a pivotal role in driving cancer cell growth and proliferation by regulating key metabolic pathways and gene expression. As a transcription factor, Nrf2 is frequently overactivated in various cancers, contributing to their aggressive nature. A key function of Nrf2 is metabolic regulation, particularly in maintaining intracellular levels of reduced glutathione (GSH), a critical antioxidant required for detoxification and oxidative stress defense. By activating enzymes involved in GSH synthesis, including G6PD, TKT, and PGD, through the pentose phosphate pathway (PPP), Nrf2 ensures a steady supply of nucleotides and amino acids essential for sustained cancer cell proliferation [151,152].

Beyond metabolism, Nrf2 exerts significant control over cell cycle progression, facilitating cancer cell division. It activates genes such as Bmpr1a, Igf1, and Pdgf-c, which drive progression through the G1 and S phases. The loss of Nrf2 induces G2/M-phase arrest, underscoring its critical role in maintaining cell cycle continuity. Additionally, Nrf2 interacts with the PI3K/AKT pathway, promoting anabolic metabolism and enhancing cellular survival through phosphorylation of key proteins, including AKT and GSK3 [153].

Mitochondrial function is another crucial aspect governed by Nrf2. It regulates mitochondrial biogenesis, substrate availability for oxidative phosphorylation, and the removal of damaged mitochondria, ensuring continuous energy production to support the high metabolic demands of proliferating cancer cells. In pancreatic cancer, Nrf2 depletion disrupts epidermal growth factor receptor (EGFR) signaling and impairs mRNA translation due to oxidative damage, highlighting its essential role in maintaining translational capacity and cellular viability [154].

Nrf2 also plays a central role in apoptosis resistance. By upregulating antioxidant enzymes such as GCL and GSR, it effectively neutralizes reactive oxygen species (ROS), shielding cancer cells from oxidative stress-induced cell death [155]. Additionally, Nrf2 suppresses apoptosis by inhibiting p53-mediated gene regulation and increasing the expression of x-CT, NQO1, and GST [156]. The Nrf2-mediated upregulation of GSTP1 further prevents proapoptotic JNK activation, while p62 induction promotes selective autophagy of Keap1, reinforcing a feedback loop that sustains apoptosis evasion [157]. Furthermore, Nrf2 enhances Bcl-2 expression, thereby reducing apoptosis in response to chemotherapeutic agents like etoposide, and suppresses TNF-induced cell death through HO-1 expression [158,159].

Angiogenesis, a hallmark of cancer progression, is also regulated by Nrf2. It promotes endothelial cell proliferation, migration, and capillary formation by upregulating HO-1, which enhances VEGF production. The VEGF/Nrf2 axis has been implicated in angiogenesis across multiple cancers, including gastric, glioma, and pancreatic tumors [121,122,123]. Inhibition of Nrf2 has been shown to suppress hypoxia-induced HIF-1α/VEGF signaling, highlighting the crosstalk between these pathways in tumor-driven angiogenesis [160].

Nrf2 also plays a key role in chemoresistance by increasing the expression of antioxidant enzymes (Trx, Prx, GCL) and phase II detoxifying enzymes (NQO1, GSTP1), thereby reducing the efficacy of chemotherapy. Downregulation of Nrf2 has been associated with increased cisplatin sensitivity, whereas its overexpression confers resistance to multiple chemotherapeutic agents [161,162,163]. Additionally, Nrf2 enhances multidrug resistance protein (MRP) expression, limiting intracellular drug accumulation and further reducing chemotherapy effectiveness [164]. Beyond chemoresistance, Nrf2 also contributes to radioresistance by upregulating antioxidant defenses, such as HO-1 and NQO1, while interacting with resistance pathways like HIF-1 and NF-κB [165,166]. Given its role in both chemoresistance and radioresistance, targeting Nrf2 presents a potential therapeutic strategy for improving cancer treatment outcomes [167].

## 7. Surgery Stress and Cancer Progression

### 7.1. Surgical Intervention and the Promotion of Micrometastatic and Residual Disease Growth

Metastatic cancer cells can disseminate from the primary tumor early in tumor progression, establishing micrometastases at distant sites that often remain clinically undetectable. These micrometastases may persist in a dormant state, balancing cell proliferation and apoptosis [168]. However, local and systemic inflammatory responses triggered by surgical trauma can disrupt this equilibrium, potentially stimulating the growth of dormant micrometastases [168]. In addition to inflammation-driven tumor growth, the surgical removal of the primary tumor may eliminate inhibitory mechanisms that previously suppressed metastatic expansion, further facilitating disease progression.

Primary tumors release both proangiogenic and antiangiogenic factors, maintaining a localized balance that favors angiogenesis due to the presence of new vasculature. However, in systemic circulation, stable antiangiogenic molecules, such as angiostatin, endostatin, and thrombospondin, are present at higher levels, suppressing neovascularization at distant sites and keeping micrometastases in a dormant state [169]. The removal of the primary tumor results in a rapid decline of these inhibitory factors, potentially triggering the “angiogenic switch” and enabling micrometastases to grow aggressively [169,170]. This process is further exacerbated by surgery-induced increases in growth factors and proangiogenic mediators, accelerating tumor expansion.

Beyond its impact on angiogenesis, surgical intervention can also facilitate immune evasion by impairing adaptive immune responses. The postoperative period is characterized by a reduction in circulating dendritic cells (DCs), which are essential for immune surveillance. Experimental studies have shown that administering DC vaccines to tumor-bearing mice can counteract the protumorigenic effects of surgery [171]. Additionally, surgical trauma is associated with a diminished Th1 immune response in humans, compromising cytotoxic T-cell activation and weakening antitumor immunity [172]. This immunosuppression can persist for several weeks postoperatively and is more prolonged following laparotomy than in minimally invasive laparoscopic procedures.

Furthermore, surgical intervention promotes neutrophil recruitment and the formation of neutrophil extracellular traps (NETs) at the surgical site. These NETs can persist for an extended period, enhancing the survival and proliferation of residual tumor cells by activating key signaling pathways such as Stat3 and NF-κB [170]. As a result, the perioperative period represents a critical window in which the immune environment becomes particularly permissive to tumor regrowth and metastatic progression.

Surgical procedures induce inflammatory responses through multiple mechanisms, including direct tissue injury and the risk of postoperative infections. These events lead to the release of inflammatory mediators and the recruitment of immune cells, such as neutrophils and monocytes [6,173]. Among these mediators, cytokines like interleukin-1 (IL-1) and tumor necrosis factor-alpha (TNF-α), along with vascular endothelial growth factor (VEGF) and MMPs, play critical roles in shaping the tumor microenvironment to promote cancer progression [6,174].

Prostaglandin E2 (PGE2), a key product of cyclooxygenase activity, is involved in various physiological and pathological processes, including cellular proliferation and angiogenesis [175]. Elevated PGE2 levels have been linked to enhanced neoplastic progression across multiple cancer types. In lung cancer, PGE2 facilitates metastasis by upregulating MMP9 mRNA expression while downregulating E-cadherin mRNA expression, thereby promoting tumor cell invasion and dissemination [176]. Additionally, PGE2 exerts potent immunosuppressive effects by expanding Treg cells, reducing the population of activated CD8^+^ T cells, and altering cytokine secretion profiles of T helper cells—mechanisms that collectively create an immune environment conducive to tumor growth [177].

In breast cancer, PGE2 has been implicated in the transition of disseminated tumor cells from dormancy to active proliferation within the bone microenvironment, highlighting its role in metastatic progression [178]. This provides a potential rationale for clinical observations in which cyclooxygenase-2 (COX-2) inhibition exhibits antineoplastic effects in certain patients with prostate or lung cancer [179].

### 7.2. Metabolic Changes Following Surgery

Surgical trauma induces a systemic stress response characterized by neuroendocrine dysregulation, primarily involving activation of the hypothalamic–pituitary–adrenal (HPA) axis and the sympathetic nervous system (SNS) [180]. This physiological adaptation leads to elevated circulating levels of stress-related hormones, such as glucocorticoids and catecholamines, which drive metabolic shifts favoring catabolism [181]. These alterations have been implicated in cancer progression.

The metabolic response triggered by stress hormones enhances the catabolism of glucose, fats, and proteins. Evolutionarily, this adaptation likely served as a survival mechanism, allowing organisms to maintain energy levels in the absence of food until recovery processes were complete [181]. However, in the context of cancer, these metabolic alterations may facilitate disease progression. Following surgery, blood glucose levels rise, a phenomenon known as stress hyperglycemia [182]. This elevation is primarily driven by glucocorticoids (GCs) and catecholamines (CAs), which stimulate hepatic glycogenolysis and gluconeogenesis [181]. Elevated glucose concentrations have been linked to tumor progression through multiple mechanisms, including increased tumor cell proliferation [183], invasion [184], migration [185], resistance [186], and decreased chemotherapy sensitivity [187]. For instance, hyperglycemia has been shown to accelerate pancreatic cancer progression, accompanied by increased phosphorylation of STAT3 and upregulation of MYC [188]. Additionally, stress hyperglycemia shares several characteristics with type 2 diabetes, such as heightened oxidative stress and activation of stress-responsive kinases [189]. Elevated blood glucose levels oxidative stress [190], which promotes cancer progression by inducing DNA mutations, causing DNA damage, increasing genome instability, and stimulating uncontrolled cell proliferation. Hormonal fluctuations during surgery also trigger the breakdown of fats and proteins. Increased levels of free fatty acids contribute to oxidative stress by enhancing mitochondrial ROS production and activating NADPH oxidase in tissues and macrophages. This oxidative imbalance further accelerates tumor progression through the previously described mechanisms [189].

## 8. Role of Anti-Inflammatory Drugs in Cancer Prevention and Treatment

Recent advances in cancer research have highlighted the critical role of inflammation in tumor initiation, progression, and metastasis. These findings have spurred interest in anti-inflammatory drugs as potential anticancer therapies aimed at targeting the inflammatory tumor microenvironment.

### 8.1. Efficacy of Anti-Inflammatory Agents

A key advantage of targeting the inflammatory microenvironment is the relative genomic and epigenetic stability of immune cells compared to the highly mutable and treatment-resistant nature of cancer cells. This stability may improve the efficacy of anti-inflammatory therapies, potentially overcoming some of the drug resistance challenges commonly associated with conventional cytotoxic treatments [77,191]. However, anti-inflammatory agents typically do not exert direct cytotoxic effects on tumor cells. As a result, their therapeutic efficacy is often optimized when used in combination with standard treatments, such as chemotherapy or radiation therapy, underscoring the importance of integrative treatment strategies in cancer management.

Several anti-inflammatory agents, including cyclooxygenase-2 (COX-2) inhibitors, aspirin, and corticosteroids like dexamethasone, have shown efficacy in reducing tumor incidence when administered prophylactically [192]. For instance, aspirin has been found to lower the risk of colorectal cancer [193] and is associated with a reduced incidence of breast and prostate cancers [194,195,196], particularly in individuals with specific genetic predispositions. Despite these benefits, the widespread clinical use of these agents remains limited due to their potential adverse effects, restricting their application primarily to high-risk populations. Identifying and screening such high-risk individuals may enhance the precision and effectiveness of these therapeutic interventions [197].

### 8.2. Anti-Inflammatory Therapies Targeting Protumorigenic Inflammation

Anti-inflammatory therapies target various aspects of protumorigenic inflammation [198]: (1) inhibiting signaling pathways and transcription factors activated by inflammatory cytokines that promote tumor cell survival and growth; (2) suppressing chemokines and cytokines that recruit and sustain inflammatory cells within the tumor microenvironment; (3) modulating inflammation induced by cancer treatment to prevent tumor recurrence; (4) depleting immune and inflammatory cells that facilitate tumor growth while preserving those that support protective immune responses; and (5) selectively inhibiting tumor-promoting cytokines without disrupting the expression of antitumorigenic cytokines.

In certain cases, monotherapies effectively target inflammation. For example, lymphoid malignancies driven by constitutive NF-κB or STAT3 activation may respond favorably to inhibitors targeting these pathways. However, in most cases, anti-inflammatory therapies demonstrate greater efficacy when combined with conventional treatments. Notably, chemotherapeutic agents that activate NF-κB can induce resistance to subsequent therapies, emphasizing the potential of combining NF-κB inhibitors with genotoxic agents to overcome treatment resistance [144,199]. Nonetheless, prolonged NF-κB inhibition may lead to extensive immune suppression and exacerbated inflammation, complicating its clinical application [9,149].

Cytokine inhibitors represent a promising class of anti-inflammatory agents. Therapeutics targeting proinflammatory cytokines, such as TNF-α and IL-6, are currently under evaluation in clinical trials [114]. While these treatments do not directly induce cytotoxicity in cancer cells, they have the potential to stabilize disease progression or achieve partial responses. Notably, early-phase clinical trials suggest that anticytokine therapies can enhance therapeutic outcomes when combined with conventional treatments, such as chemotherapy [114,200]. Additionally, antichemokine therapies targeting chemokine receptors—including CCR2, CCR4, and CXCR4—are actively being investigated [114,200]. These treatments aim to disrupt the recruitment of inflammatory cells that support tumor progression and metastasis. Moreover, therapies targeting IL-1β have shown significant potential in malignancies such as multiple myeloma, where they inhibit tumor growth and delay progression to more aggressive disease stages [149].

### 8.3. Emerging Therapies and the Role of STING Agonists

The cGAS–STING pathway serves as a crucial sensor of cytosolic DNA, triggering the production of type I interferons and various inflammatory cytokines. This activation plays a fundamental role in innate immune defense, particularly against viral and bacterial infections [201,202]. Given its role in immune activation, STING has emerged as a promising target for cancer immunotherapy. STING agonists are currently being explored for their ability to stimulate antitumor immunity. Preclinical studies indicate that compounds like cGAMP enhance immune responses by promoting cytokine production and increasing immune cell infiltration into tumors. Furthermore, STING agonists can enhance the efficacy of chemotherapy and radiotherapy by improving immune recognition of tumor cells [203].

Another promising strategy involves combining STING agonists with immune checkpoint inhibitors, such as anti-PD-1 and anti-PD-L1 antibodies. These combinations have demonstrated the ability to enhance tumor regression by converting poorly immunogenic “cold” tumors into “hot” tumors with increased immune activity [204]. Ongoing clinical trials are currently assessing the safety and efficacy of STING-based therapies. While challenges remain, including the risk of inflammatory toxicity, STING agonists hold significant potential as a novel approach to improving cancer treatment outcomes [204].

## 9. Conclusions

The intricate relationship between inflammation and cancer progression is now well established, influencing tumor initiation, promotion, metastasis, and therapy resistance. Inflammation-driven genetic instability, epigenetic modifications, and cytokine signaling contribute to a tumor-supportive microenvironment, fostering malignant transformation. Chronic inflammation accelerates tumor progression by promoting angiogenesis, immune evasion, and metabolic reprogramming, which sustain tumor growth and facilitate metastasis. Additionally, inflammatory cytokines such as TNF-α, IL-6, and IL-23 activate key oncogenic pathways, including NF-κB and STAT3, reinforcing a protumorigenic inflammatory loop.

Inflammation also plays a central role in the metastatic cascade. Cytokines, chemokines, and immune cells establish a premetastatic niche that enhances tumor cell survival, extravasation, and colonization. Hypoxia-induced immune suppression, along with tumor-associated macrophages and Tregs, further accelerates metastatic progression. Furthermore, inflammation significantly contributes to therapy resistance, as proinflammatory responses following chemotherapy or radiation therapy can promote tumor recurrence by activating survival pathways and altering the tumor microenvironment.

Given its central role in cancer pathophysiology, targeting inflammation presents a promising therapeutic strategy. Anti-inflammatory agents, such as COX-2 inhibitors and cytokine blockers, have shown potential in reducing tumor progression and enhancing treatment efficacy. Additionally, emerging immunotherapies, including STING agonists and immune checkpoint inhibitors, provide new avenues for modulating inflammation while simultaneously strengthening antitumor immunity.

Future research should prioritize refining inflammation-targeted therapies, identifying patient-specific inflammatory profiles, and integrating these strategies into existing treatment regimens. A deeper understanding of inflammation’s dual role—both as a driver of cancer progression and as a key component of immune surveillance—will be critical in developing more effective and personalized cancer therapies.

## Data Availability

No new data were created or analyzed in this study.

## References

[1] Balkwill F., Mantovani A. (2001). Inflammation and cancer: Back to Virchow?. Lancet.

[2] Multhoff G., Molls M., Radons J. (2012). Chronic Inflammation in Cancer Development. Front. Immunol..

[3] Mantovani A. (2005). Cancer: Inflammation by remote control. Nature.

[4] Thun M.J., DeLancey J.O., Center M.M., Jemal A., Ward E.M. (2010). The global burden of cancer: Priorities for prevention. Carcinogenesis.

[5] Clinton S.K., Giovannucci E.L., Hursting S.D. (2020). The World Cancer Research Fund/American Institute for Cancer Research Third Expert Report on Diet, Nutrition, Physical Activity, and Cancer: Impact and Future Directions. J. Nutr..

[6] Coussens L.M., Werb Z. (2002). Inflammation and cancer. Nature.

[7] Mantovani A., Allavena P., Sica A., Balkwill F. (2008). Cancer-related inflammation. Nature.

[8] Wang X., He J., Pan J. (2023). Editorial: Dual role of inflammatory mediators in cancer immunotherapy. Front. Immunol..

[9] Zhao H., Wu L., Yan G., Chen Y., Zhou M., Wu Y., Li Y. (2021). Inflammation and tumor progression: Signaling pathways and targeted intervention. Signal Transduct. Target. Ther..

[10] Qin S., Jiang J., Lu Y., Nice E.C., Huang C., Zhang J., He W. (2020). Emerging role of tumor cell plasticity in modifying therapeutic response. Signal Transduct. Target. Ther..

[11] Derks L.L.M., van Boxtel R. (2023). Stem cell mutations, associated cancer risk, and consequences for regenerative medicine. Cell Stem Cell.

[12] Xiang Y., Zhang M., Jiang D., Su Q., Shi J. (2023). The role of inflammation in autoimmune disease: A therapeutic target. Front. Immunol..

[13] Morgillo F., Dallio M., Della Corte C.M., Gravina A.G., Viscardi G., Loguercio C., Ciardiello F., Federico A. (2018). Carcinogenesis as a Result of Multiple Inflammatory and Oxidative Hits: A Comprehensive Review from Tumor Microenvironment to Gut Microbiota. Neoplasia.

[14] Tafani M., Sansone L., Limana F., Arcangeli T., De Santis E., Polese M., Fini M., Russo M.A. (2016). The Interplay of Reactive Oxygen Species, Hypoxia, Inflammation, and Sirtuins in Cancer Initiation and Progression. Oxidative Med. Cell. Longev..

[15] Zhou R.W., Harpaz N., Itzkowitz S.H., Parsons R.E. (2023). Molecular mechanisms in colitis-associated colorectal cancer. Oncogenesis.

[16] Ranneh Y., Ali F., Akim A.M., Hamid H.A., Khazaai H., Fadel A. (2017). Crosstalk between reactive oxygen species and pro-inflammatory markers in developing various chronic diseases: A review. Appl. Biol. Chem..

[17] Checa J., Aran J.M. (2020). Reactive Oxygen Species: Drivers of Physiological and Pathological Processes. J. Inflamm. Res..

[18] Shimizu T., Marusawa H., Endo Y., Chiba T. (2012). Inflammation-mediated genomic instability: Roles of activation-induced cytidine deaminase in carcinogenesis. Cancer Sci..

[19] Lee D.S.W., Rojas O.L., Gommerman J.L. (2021). B cell depletion therapies in autoimmune disease: Advances and mechanistic insights. Nat. Rev. Drug Discov..

[20] Das D., Karthik N., Taneja R. (2021). Crosstalk Between Inflammatory Signaling and Methylation in Cancer. Front. Cell Dev. Biol..

[21] Cheng Y., He C., Wang M., Ma X., Mo F., Yang S., Han J., Wei X. (2019). Targeting epigenetic regulators for cancer therapy: Mechanisms and advances in clinical trials. Signal Transduct. Target. Ther..

[22] Quail D.F., Joyce J.A. (2013). Microenvironmental regulation of tumor progression and metastasis. Nat. Med..

[23] Li W., Liu Q., Shi J., Xu X., Xu J. (2023). The role of TNF-α in the fate regulation and functional reprogramming of mesenchymal stem cells in an inflammatory microenvironment. Front. Immunol..

[24] Hirano T. (2021). IL-6 in inflammation, autoimmunity and cancer. Int. Immunol..

[25] Hibino S., Kawazoe T., Kasahara H., Itoh S., Ishimoto T., Sakata-Yanagimoto M., Taniguchi K. (2021). Inflammation-Induced Tumorigenesis and Metastasis. Int. J. Mol. Sci..

[26] Briukhovetska D., Dörr J., Endres S., Libby P., Dinarello C.A., Kobold S. (2021). Interleukins in cancer: From biology to therapy. Nat. Rev. Cancer.

[27] Rose-John S., Jenkins B.J., Garbers C., Moll J.M., Scheller J. (2023). Targeting IL-6 trans-signalling: Past, present and future prospects. Nat. Rev. Immunol..

[28] Chen M., Ye X., Wang R., Poon K. (2020). Research progress of cancer stem cells and IL-6/STAT3 signaling pathway in esophageal adenocarcinoma. Transl. Cancer Res..

[29] Yang K., Wang X., Zhang H., Wang Z., Nan G., Li Y., Zhang F., Mohammed M.K., Haydon R.C., Luu H.H. (2016). The evolving roles of canonical WNT signaling in stem cells and tumorigenesis: Implications in targeted cancer therapies. Lab. Investig..

[30] Kawanishi S., Ohnishi S., Ma N., Hiraku Y., Murata M. (2017). Crosstalk between DNA Damage and Inflammation in the Multiple Steps of Carcinogenesis. Int. J. Mol. Sci..

[31] Grivennikov S.I., Karin M. (2010). Inflammation and oncogenesis: A vicious connection. Curr. Opin. Genet. Dev..

[32] Catanzaro J.M., Sheshadri N., Pan J.A., Sun Y., Shi C., Li J., Powers R.S., Crawford H.C., Zong W.X. (2014). Oncogenic Ras induces inflammatory cytokine production by upregulating the squamous cell carcinoma antigens SerpinB3/B4. Nat. Commun..

[33] Greten F.R., Grivennikov S.I. (2019). Inflammation and Cancer: Triggers, Mechanisms, and Consequences. Immunity.

[34] Quante M., Varga J., Wang T.C., Greten F.R. (2013). The Gastrointestinal Tumor Microenvironment. Gastroenterology.

[35] Turizo-Smith A.D., Córdoba-Hernandez S., Mejía-Guarnizo L.V., Monroy-Camacho P.S., Rodríguez-García J.A. (2024). Inflammation and cancer: Friend or foe?. Front. Pharmacol..

[36] Li X., Yang Y., Zhang B., Lin X., Fu X., An Y., Zou Y., Wang J.-X., Wang Z., Yu T. (2022). Lactate metabolism in human health and disease. Signal Transduct. Target. Ther..

[37] Fedele M., Sgarra R., Battista S., Cerchia L., Manfioletti G. (2022). The Epithelial-Mesenchymal Transition at the Crossroads between Metabolism and Tumor Progression. Int. J. Mol. Sci..

[38] El Tekle G., Garrett W.S. (2023). Bacteria in cancer initiation, promotion and progression. Nat. Rev. Cancer.

[39] de Visser K.E., Joyce J.A. (2023). The evolving tumor microenvironment: From cancer initiation to metastatic outgrowth. Cancer Cell.

[40] Semenza G.L. (2010). Defining the role of hypoxia-inducible factor 1 in cancer biology and therapeutics. Oncogene.

[41] Chouaib S., Noman M.Z., Kosmatopoulos K., Curran M.A. (2017). Hypoxic stress: Obstacles and opportunities for innovative immunotherapy of cancer. Oncogene.

[42] Labani-Motlagh A., Ashja-Mahdavi M., Loskog A. (2020). The Tumor Microenvironment: A Milieu Hindering and Obstructing Antitumor Immune Responses. Front. Immunol..

[43] Noman M.Z., Hasmim M., Messai Y., Terry S., Kieda C., Janji B., Chouaib S. (2015). Hypoxia: A key player in antitumor immune response. A Review in the Theme: Cellular Responses to Hypoxia. Am. J. Physiol. Cell Physiol..

[44] Schito L., Semenza G.L. (2016). Hypoxia-Inducible Factors: Master Regulators of Cancer Progression. Trends Cancer.

[45] Lugano R., Ramachandran M., Dimberg A. (2020). Tumor angiogenesis: Causes, consequences, challenges and opportunities. Cell. Mol. Life Sci..

[46] Liao D., Johnson R.S. (2007). Hypoxia: A key regulator of angiogenesis in cancer. Cancer Metastasis Rev..

[47] Ferrara N., Gerber H.P., LeCouter J. (2003). The biology of VEGF and its receptors. Nat. Med..

[48] van Hinsbergh V.W., Koolwijk P. (2008). Endothelial sprouting and angiogenesis: Matrix metalloproteinases in the lead. Cardiovasc. Res..

[49] Lamalice L., Le Boeuf F., Huot J. (2007). Endothelial cell migration during angiogenesis. Circ. Res..

[50] Weis S.M., Cheresh D.A. (2005). Pathophysiological consequences of VEGF-induced vascular permeability. Nature.

[51] Azzi S., Hebda J.K., Gavard J. (2013). Vascular permeability and drug delivery in cancers. Front. Oncol..

[52] Carmeliet P., Jain R.K. (2011). Principles and mechanisms of vessel normalization for cancer and other angiogenic diseases. Nat. Rev. Drug Discov..

[53] Schito L. (2019). Hypoxia-Dependent Angiogenesis and Lymphangiogenesis in Cancer. Adv. Exp. Med. Biol..

[54] Joyce J.A., Pollard J.W. (2009). Microenvironmental regulation of metastasis. Nat. Rev. Cancer.

[55] Kujawski M., Kortylewski M., Lee H., Herrmann A., Kay H., Yu H. (2008). Stat3 mediates myeloid cell-dependent tumor angiogenesis in mice. J. Clin. Investig..

[56] Dang E.V., Barbi J., Yang H.Y., Jinasena D., Yu H., Zheng Y., Bordman Z., Fu J., Kim Y., Yen H.R. (2011). Control of T(H)17/T(reg) balance by hypoxia-inducible factor 1. Cell.

[57] Noman M.Z., Desantis G., Janji B., Hasmim M., Karray S., Dessen P., Bronte V., Chouaib S. (2014). PD-L1 is a novel direct target of HIF-1α, and its blockade under hypoxia enhanced MDSC-mediated T cell activation. J. Exp. Med..

[58] Corzo C.A., Condamine T., Lu L., Cotter M.J., Youn J.I., Cheng P., Cho H.I., Celis E., Quiceno D.G., Padhya T. (2010). HIF-1α regulates function and differentiation of myeloid-derived suppressor cells in the tumor microenvironment. J. Exp. Med..

[59] Chaturvedi P., Gilkes D.M., Takano N., Semenza G.L. (2014). Hypoxia-inducible factor-dependent signaling between triple-negative breast cancer cells and mesenchymal stem cells promotes macrophage recruitment. Proc. Natl. Acad. Sci. USA.

[60] Werno C., Menrad H., Weigert A., Dehne N., Goerdt S., Schledzewski K., Kzhyshkowska J., Brüne B. (2010). Knockout of HIF-1α in tumor-associated macrophages enhances M2 polarization and attenuates their pro-angiogenic responses. Carcinogenesis.

[61] White J.R., Harris R.A., Lee S.R., Craigon M.H., Binley K., Price T., Beard G.L., Mundy C.R., Naylor S. (2004). Genetic amplification of the transcriptional response to hypoxia as a novel means of identifying regulators of angiogenesis. Genomics.

[62] Gabrilovich D.I., Chen H.L., Girgis K.R., Cunningham H.T., Meny G.M., Nadaf S., Kavanaugh D., Carbone D.P. (1996). Production of vascular endothelial growth factor by human tumors inhibits the functional maturation of dendritic cells. Nat. Med..

[63] Mancino A., Schioppa T., Larghi P., Pasqualini F., Nebuloni M., Chen I.H., Sozzani S., Austyn J.M., Mantovani A., Sica A. (2008). Divergent effects of hypoxia on dendritic cell functions. Blood.

[64] Clambey E.T., McNamee E.N., Westrich J.A., Glover L.E., Campbell E.L., Jedlicka P., de Zoeten E.F., Cambier J.C., Stenmark K.R., Colgan S.P. (2012). Hypoxia-inducible factor-1 alpha-dependent induction of FoxP3 drives regulatory T-cell abundance and function during inflammatory hypoxia of the mucosa. Proc. Natl. Acad. Sci. USA.

[65] Facciabene A., Peng X., Hagemann I.S., Balint K., Barchetti A., Wang L.P., Gimotty P.A., Gilks C.B., Lal P., Zhang L. (2011). Tumour hypoxia promotes tolerance and angiogenesis via CCL28 and T(reg) cells. Nature.

[66] Hasmim M., Noman M.Z., Messai Y., Bordereaux D., Gros G., Baud V., Chouaib S. (2013). Cutting edge: Hypoxia-induced Nanog favors the intratumoral infiltration of regulatory T cells and macrophages via direct regulation of TGF-β1. J. Immunol..

[67] Almand B., Clark J.I., Nikitina E., van Beynen J., English N.R., Knight S.C., Carbone D.P., Gabrilovich D.I. (2001). Increased production of immature myeloid cells in cancer patients: A mechanism of immunosuppression in cancer. J. Immunol..

[68] Belhabib I., Zaghdoudi S., Lac C., Bousquet C., Jean C. (2021). Extracellular Matrices and Cancer-Associated Fibroblasts: Targets for Cancer Diagnosis and Therapy?. Cancers.

[69] Siska P.J., Singer K., Evert K., Renner K., Kreutz M. (2020). The immunological Warburg effect: Can a metabolic-tumor-stroma score (MeTS) guide cancer immunotherapy?. Immunol. Rev..

[70] Burns J.S., Manda G. (2017). Metabolic Pathways of the Warburg Effect in Health and Disease: Perspectives of Choice, Chain or Chance. Int. J. Mol. Sci..

[71] Papa S., Choy P.M., Bubici C. (2019). The ERK and JNK pathways in the regulation of metabolic reprogramming. Oncogene.

[72] Kim J., DeBerardinis R.J. (2019). Mechanisms and Implications of Metabolic Heterogeneity in Cancer. Cell Metab..

[73] Li S., Yuan H., Li L., Li Q., Lin P., Li K. (2025). Oxidative Stress and Reprogramming of Lipid Metabolism in Cancers. Antioxidants.

[74] Mimeault M., Batra S.K. (2013). Hypoxia-inducing factors as master regulators of stemness properties and altered metabolism of cancer- and metastasis-initiating cells. J. Cell. Mol. Med..

[75] Wu Y., Pu X., Wang X., Xu M. (2024). Reprogramming of lipid metabolism in the tumor microenvironment: A strategy for tumor immunotherapy. Lipids Health Dis..

[76] Chen S., Zhu H., Jounaidi Y. (2024). Comprehensive snapshots of natural killer cells functions, signaling, molecular mechanisms and clinical utilization. Signal Transduct. Target. Ther..

[77] Grivennikov S.I., Greten F.R., Karin M. (2010). Immunity, Inflammation, and Cancer. Cell.

[78] Hernandez C., Huebener P., Schwabe R.F. (2016). Damage-associated molecular patterns in cancer: A double-edged sword. Oncogene.

[79] Ashrafizadeh M., Farhood B., Eleojo Musa A., Taeb S., Najafi M. (2020). Damage-associated molecular patterns in tumor radiotherapy. Int. Immunopharmacol..

[80] Roehlecke C., Schmidt M.H.H. (2020). Tunneling Nanotubes and Tumor Microtubes in Cancer. Cancers.

[81] Sato A., Rahman N.I.A., Shimizu A., Ogita H. (2021). Cell-to-cell contact-mediated regulation of tumor behavior in the tumor microenvironment. Cancer Sci..

[82] Lowery L.A., Van Vactor D. (2009). The trip of the tip: Understanding the growth cone machinery. Nat. Rev. Mol. Cell Biol..

[83] Osswald M., Jung E., Sahm F., Solecki G., Venkataramani V., Blaes J., Weil S., Horstmann H., Wiestler B., Syed M. (2015). Brain tumour cells interconnect to a functional and resistant network. Nature.

[84] Jung E., Osswald M., Blaes J., Wiestler B., Sahm F., Schmenger T., Solecki G., Deumelandt K., Kurz F.T., Xie R. (2017). Tweety-Homolog 1 Drives Brain Colonization of Gliomas. J. Neurosci..

[85] Melwani P.K., Pandey B.N. (2023). Tunneling nanotubes: The intercellular conduits contributing to cancer pathogenesis and its therapy. Biochim. Biophys. Acta Rev. Cancer.

[86] Lou E., Fujisawa S., Morozov A., Barlas A., Romin Y., Dogan Y., Gholami S., Moreira A.L., Manova-Todorova K., Moore M.A. (2012). Tunneling nanotubes provide a unique conduit for intercellular transfer of cellular contents in human malignant pleural mesothelioma. PLoS ONE.

[87] Seyfried T.N., Huysentruyt L.C. (2013). On the origin of cancer metastasis. Crit. Rev. Oncog..

[88] Fares J., Fares M.Y., Khachfe H.H., Salhab H.A., Fares Y. (2020). Molecular principles of metastasis: A hallmark of cancer revisited. Signal Transduct. Target. Ther..

[89] Cao X. (2016). Self-regulation and cross-regulation of pattern-recognition receptor signalling in health and disease. Nat. Rev. Immunol..

[90] Mittal V. (2018). Epithelial Mesenchymal Transition in Tumor Metastasis. Annu. Rev. Pathol..

[91] Heerboth S., Housman G., Leary M., Longacre M., Byler S., Lapinska K., Willbanks A., Sarkar S. (2015). EMT and tumor metastasis. Clin. Transl. Med..

[92] Aiello N.M., Kang Y. (2019). Context-dependent EMT programs in cancer metastasis. J. Exp. Med..

[93] Lamouille S., Xu J., Derynck R. (2014). Molecular mechanisms of epithelial-mesenchymal transition. Nat. Rev. Mol. Cell Biol..

[94] Thiery J.P., Acloque H., Huang R.Y., Nieto M.A. (2009). Epithelial-mesenchymal transitions in development and disease. Cell.

[95] Peinado H., Olmeda D., Cano A. (2007). Snail, Zeb and bHLH factors in tumour progression: An alliance against the epithelial phenotype?. Nat. Rev. Cancer.

[96] López-Novoa J.M., Nieto M.A. (2009). Inflammation and EMT: An alliance towards organ fibrosis and cancer progression. EMBO Mol. Med..

[97] Bates R.C., Mercurio A.M. (2003). Tumor necrosis factor-alpha stimulates the epithelial-to-mesenchymal transition of human colonic organoids. Mol. Biol. Cell.

[98] Ricciardi M., Zanotto M., Malpeli G., Bassi G., Perbellini O., Chilosi M., Bifari F., Krampera M. (2015). Epithelial-to-mesenchymal transition (EMT) induced by inflammatory priming elicits mesenchymal stromal cell-like immune-modulatory properties in cancer cells. Br. J. Cancer.

[99] Zhu Y., Cheng Y., Guo Y., Chen J., Chen F., Luo R., Li A. (2016). Protein kinase D2 contributes to TNF-α-induced epithelial mesenchymal transition and invasion via the PI3K/GSK-3β/β-catenin pathway in hepatocellular carcinoma. Oncotarget.

[100] Cohen E.N., Gao H., Anfossi S., Mego M., Reddy N.G., Debeb B., Giordano A., Tin S., Wu Q., Garza R.J. (2015). Inflammation Mediated Metastasis: Immune Induced Epithelial-To-Mesenchymal Transition in Inflammatory Breast Cancer Cells. PLoS ONE.

[101] Song J., Feng L., Zhong R., Xia Z., Zhang L., Cui L., Yan H., Jia X., Zhang Z. (2017). Icariside II inhibits the EMT of NSCLC cells in inflammatory microenvironment via down-regulation of Akt/NF-κB signaling pathway. Mol. Carcinog..

[102] Lv N., Gao Y., Guan H., Wu D., Ding S., Teng W., Shan Z. (2015). Inflammatory mediators, tumor necrosis factor-α and interferon-γ, induce EMT in human PTC cell lines. Oncol. Lett..

[103] Sun K.H., Sun G.H., Wu Y.C., Ko B.J., Hsu H.T., Wu S.T. (2016). TNF-α augments CXCR2 and CXCR3 to promote progression of renal cell carcinoma. J. Cell. Mol. Med..

[104] Latorre E., Tebaldi T., Viero G., Spartà A.M., Quattrone A., Provenzani A. (2012). Downregulation of HuR as a new mechanism of doxorubicin resistance in breast cancer cells. Mol. Cancer.

[105] Sullivan N.J., Sasser A.K., Axel A.E., Vesuna F., Raman V., Ramirez N., Oberyszyn T.M., Hall B.M. (2009). Interleukin-6 induces an epithelial-mesenchymal transition phenotype in human breast cancer cells. Oncogene.

[106] Colomiere M., Ward A.C., Riley C., Trenerry M.K., Cameron-Smith D., Findlay J., Ackland L., Ahmed N. (2009). Cross talk of signals between EGFR and IL-6R through JAK2/STAT3 mediate epithelial-mesenchymal transition in ovarian carcinomas. Br. J. Cancer.

[107] Suarez-Carmona M., Bourcy M., Lesage J., Leroi N., Syne L., Blacher S., Hubert P., Erpicum C., Foidart J.M., Delvenne P. (2015). Soluble factors regulated by epithelial-mesenchymal transition mediate tumour angiogenesis and myeloid cell recruitment. J. Pathol..

[108] Lim S., Becker A., Zimmer A., Lu J., Buettner R., Kirfel J. (2013). SNAI1-mediated epithelial-mesenchymal transition confers chemoresistance and cellular plasticity by regulating genes involved in cell death and stem cell maintenance. PLoS ONE.

[109] Chen D., Li W., Liu S., Su Y., Han G., Xu C., Liu H., Zheng T., Zhou Y., Mao C. (2015). Interleukin-23 promotes the epithelial-mesenchymal transition of oesophageal carcinoma cells via the Wnt/β-catenin pathway. Sci. Rep..

[110] Zhang Q., Liu S., Parajuli K.R., Zhang W., Zhang K., Mo Z., Liu J., Chen Z., Yang S., Wang A.R. (2017). Interleukin-17 promotes prostate cancer via MMP7-induced epithelial-to-mesenchymal transition. Oncogene.

[111] Jiang Y.X., Yang S.W., Li P.A., Luo X., Li Z.Y., Hao Y.X., Yu P.W. (2017). The promotion of the transformation of quiescent gastric cancer stem cells by IL-17 and the underlying mechanisms. Oncogene.

[112] Elgundi Z., Papanicolaou M., Major G., Cox T.R., Melrose J., Whitelock J.M., Farrugia B.L. (2019). Cancer Metastasis: The Role of the Extracellular Matrix and the Heparan Sulfate Proteoglycan Perlecan. Front. Oncol..

[113] Takata F., Nakagawa S., Matsumoto J., Dohgu S. (2021). Blood-Brain Barrier Dysfunction Amplifies the Development of Neuroinflammation: Understanding of Cellular Events in Brain Microvascular Endothelial Cells for Prevention and Treatment of BBB Dysfunction. Front. Cell Neurosci..

[114] Yi M., Li T., Niu M., Zhang H., Wu Y., Wu K., Dai Z. (2024). Targeting cytokine and chemokine signaling pathways for cancer therapy. Signal Transduct. Target. Ther..

[115] Hamidi H., Ivaska J. (2018). Every step of the way: Integrins in cancer progression and metastasis. Nat. Rev. Cancer.

[116] Padmanaban V., Krol I., Suhail Y., Szczerba B.M., Aceto N., Bader J.S., Ewald A.J. (2019). E-cadherin is required for metastasis in multiple models of breast cancer. Nature.

[117] Mazzone M., Bergers G. (2019). Regulation of Blood and Lymphatic Vessels by Immune Cells in Tumors and Metastasis. Annu. Rev. Physiol..

[118] Gu X., Wei S., Lv X. (2024). Circulating tumor cells: From new biological insights to clinical practice. Signal Transduct. Target. Ther..

[119] Aceto N., Bardia A., Miyamoto D.T., Donaldson M.C., Wittner B.S., Spencer J.A., Yu M., Pely A., Engstrom A., Zhu H. (2014). Circulating tumor cell clusters are oligoclonal precursors of breast cancer metastasis. Cell.

[120] Yu M., Bardia A., Wittner B.S., Stott S.L., Smas M.E., Ting D.T., Isakoff S.J., Ciciliano J.C., Wells M.N., Shah A.M. (2013). Circulating breast tumor cells exhibit dynamic changes in epithelial and mesenchymal composition. Science.

[121] Leach J., Morton J.P., Sansom O.J. (2019). Neutrophils: Homing in on the myeloid mechanisms of metastasis. Mol. Immunol..

[122] Gaertner F., Massberg S. (2019). Patrolling the vascular borders: Platelets in immunity to infection and cancer. Nat. Rev. Immunol..

[123] Schlesinger M. (2018). Role of platelets and platelet receptors in cancer metastasis. J. Hematol. Oncol..

[124] Stark K., Schubert I., Joshi U., Kilani B., Hoseinpour P., Thakur M., Grünauer P., Pfeiler S., Schmidergall T., Stockhausen S. (2018). Distinct Pathogenesis of Pancreatic Cancer Microvesicle–Associated Venous Thrombosis Identifies New Antithrombotic Targets In Vivo. Arter. Thromb. Vasc. Biol..

[125] Labelle M., Begum S., Hynes R.O. (2011). Direct signaling between platelets and cancer cells induces an epithelial-mesenchymal-like transition and promotes metastasis. Cancer Cell.

[126] Haemmerle M., Taylor M.L., Gutschner T., Pradeep S., Cho M.S., Sheng J., Lyons Y.M., Nagaraja A.S., Dood R.L., Wen Y. (2017). Platelets reduce anoikis and promote metastasis by activating YAP1 signaling. Nat. Commun..

[127] Liu Y., Cao X. (2016). Characteristics and Significance of the Pre-metastatic Niche. Cancer Cell.

[128] Kitamura T., Qian B.-Z., Pollard J.W. (2015). Immune cell promotion of metastasis. Nat. Rev. Immunol..

[129] Liu Y., Cao X. (2016). Immunosuppressive cells in tumor immune escape and metastasis. J. Mol. Med..

[130] Giles A.J., Reid C.M., Evans J.D., Murgai M., Vicioso Y., Highfill S.L., Kasai M., Vahdat L., Mackall C.L., Lyden D. (2016). Activation of Hematopoietic Stem/Progenitor Cells Promotes Immunosuppression Within the Pre–metastatic Niche. Cancer Res..

[131] Yamamura Y., Asai N., Enomoto A., Kato T., Mii S., Kondo Y., Ushida K., Niimi K., Tsunoda N., Nagino M. (2015). Akt–Girdin Signaling in Cancer-Associated Fibroblasts Contributes to Tumor Progression. Cancer Res..

[132] Hiratsuka S., Watanabe A., Sakurai Y., Akashi-Takamura S., Ishibashi S., Miyake K., Shibuya M., Akira S., Aburatani H., Maru Y. (2008). The S100A8–serum amyloid A3–TLR4 paracrine cascade establishes a pre-metastatic phase. Nat. Cell Biol..

[133] Tomita T., Sakurai Y., Ishibashi S., Maru Y. (2011). Imbalance of Clara cell-mediated homeostatic inflammation is involved in lung metastasis. Oncogene.

[134] Hansen M.T., Forst B., Cremers N., Quagliata L., Ambartsumian N., Grum-Schwensen B., Klingelhöfer J., Abdul-Al A., Herrmann P., Osterland M. (2015). A link between inflammation and metastasis: Serum amyloid A1 and A3 induce metastasis, and are targets of metastasis-inducing S100A4. Oncogene.

[135] Wculek S.K., Malanchi I. (2015). Neutrophils support lung colonization of metastasis-initiating breast cancer cells. Nature.

[136] Yan H.H., Pickup M., Pang Y., Gorska A.E., Li Z., Chytil A., Geng Y., Gray J.W., Moses H.L., Yang L. (2010). Gr-1+CD11b+ Myeloid Cells Tip the Balance of Immune Protection to Tumor Promotion in the Premetastatic Lung. Cancer Res..

[137] Rutkowski M.R., Stephen T.L., Svoronos N., Allegrezza M.J., Tesone A.J., Perales-Puchalt A., Brencicova E., Escovar-Fadul X., Nguyen J.M., Cadungog M.G. (2015). Microbially Driven TLR5-Dependent Signaling Governs Distal Malignant Progression through Tumor-Promoting Inflammation. Cancer Cell.

[138] Onal S., Turker-Burhan M., Bati-Ayaz G., Yanik H., Pesen-Okvur D. (2021). Breast cancer cells and macrophages in a paracrine-juxtacrine loop. Biomaterials.

[139] Raskov H., Orhan A., Christensen J.P., Gögenur I. (2021). Cytotoxic CD8+ T cells in cancer and cancer immunotherapy. Br. J. Cancer.

[140] Yang L., Pang Y., Moses H.L. (2010). TGF-beta and immune cells: An important regulatory axis in the tumor microenvironment and progression. Trends Immunol..

[141] He X., Xu C. (2020). Immune checkpoint signaling and cancer immunotherapy. Cell Res..

[142] Wang J., Li D., Cang H., Guo B. (2019). Crosstalk between cancer and immune cells: Role of tumor-associated macrophages in the tumor microenvironment. Cancer Med..

[143] Montfort A., Colacios C., Levade T., Andrieu-Abadie N., Meyer N., Ségui B. (2019). The TNF Paradox in Cancer Progression and Immunotherapy. Front. Immunol..

[144] Guo Q., Jin Y., Chen X., Ye X., Shen X., Lin M., Zeng C., Zhou T., Zhang J. (2024). NF-κB in biology and targeted therapy: New insights and translational implications. Signal Transduct. Target. Ther..

[145] Grivennikov S.I., Karin M. (2010). Dangerous liaisons: STAT3 and NF-kappaB collaboration and crosstalk in cancer. Cytokine Growth Factor Rev..

[146] Huang B., Lang X., Li X. (2022). The role of IL-6/JAK2/STAT3 signaling pathway in cancers. Front. Oncol..

[147] Pan Y., Yu Y., Wang X., Zhang T. (2020). Tumor-Associated Macrophages in Tumor Immunity. Front. Immunol..

[148] Li X., Zhong J., Deng X., Guo X., Lu Y., Lin J., Huang X., Wang C. (2021). Targeting Myeloid-Derived Suppressor Cells to Enhance the Antitumor Efficacy of Immune Checkpoint Blockade Therapy. Front. Immunol..

[149] Kartikasari A.E.R., Huertas C.S., Mitchell A., Plebanski M. (2021). Tumor-Induced Inflammatory Cytokines and the Emerging Diagnostic Devices for Cancer Detection and Prognosis. Front. Oncol..

[150] Liu Z.-L., Chen H.-H., Zheng L.-L., Sun L.-P., Shi L. (2023). Angiogenic signaling pathways and anti-angiogenic therapy for cancer. Signal Transduct. Target. Ther..

[151] Mitsuishi Y., Taguchi K., Kawatani Y., Shibata T., Nukiwa T., Aburatani H., Yamamoto M., Motohashi H. (2012). Nrf2 redirects glucose and glutamine into anabolic pathways in metabolic reprogramming. Cancer Cell.

[152] Wu K.C., Cui J.Y., Klaassen C.D. (2011). Beneficial role of Nrf2 in regulating NADPH generation and consumption. Toxicol. Sci..

[153] Beyer T.A., Xu W., Teupser D., auf dem Keller U., Bugnon P., Hildt E., Thiery J., Kan Y.W., Werner S. (2008). Impaired liver regeneration in Nrf2 knockout mice: Role of ROS-mediated insulin/IGF-1 resistance. EMBO J..

[154] Holmström K.M., Baird L., Zhang Y., Hargreaves I., Chalasani A., Land J.M., Stanyer L., Yamamoto M., Dinkova-Kostova A.T., Abramov A.Y. (2013). Nrf2 impacts cellular bioenergetics by controlling substrate availability for mitochondrial respiration. Biol. Open.

[155] Wild A.C., Moinova H.R., Mulcahy R.T. (1999). Regulation of gamma-glutamylcysteine synthetase subunit gene expression by the transcription factor Nrf2. J. Biol. Chem..

[156] Faraonio R., Vergara P., Di Marzo D., Pierantoni M.G., Napolitano M., Russo T., Cimino F. (2006). p53 suppresses the Nrf2-dependent transcription of antioxidant response genes. J. Biol. Chem..

[157] Elsby R., Kitteringham N.R., Goldring C.E., Lovatt C.A., Chamberlain M., Henderson C.J., Wolf C.R., Park B.K. (2003). Increased constitutive c-Jun N-terminal kinase signaling in mice lacking glutathione S-transferase Pi. J. Biol. Chem..

[158] Rushworth S.A., MacEwan D.J. (2008). HO-1 underlies resistance of AML cells to TNF-induced apoptosis. Blood.

[159] Niture S.K., Jaiswal A.K. (2012). Nrf2 protein up-regulates antiapoptotic protein Bcl-2 and prevents cellular apoptosis. J. Biol. Chem..

[160] Kim T.H., Hur E.G., Kang S.J., Kim J.A., Thapa D., Lee Y.M., Ku S.K., Jung Y., Kwak M.K. (2011). NRF2 blockade suppresses colon tumor angiogenesis by inhibiting hypoxia-induced activation of HIF-1α. Cancer Res..

[161] Kaur T., Khanduja K.L., Gupta R., Gupta N.M., Vaiphei K. (2008). Changes in antioxidant defense status in response to cisplatin and 5-FU in esophageal carcinoma. Dis. Esophagus.

[162] Ban N., Takahashi Y., Takayama T., Kura T., Katahira T., Sakamaki S., Niitsu Y. (1996). Transfection of glutathione S-transferase (GST)-pi antisense complementary DNA increases the sensitivity of a colon cancer cell line to adriamycin, cisplatin, melphalan, and etoposide. Cancer Res..

[163] Cho J.M., Manandhar S., Lee H.R., Park H.M., Kwak M.K. (2008). Role of the Nrf2-antioxidant system in cytotoxicity mediated by anticancer cisplatin: Implication to cancer cell resistance. Cancer Lett..

[164] Aleksunes L.M., Slitt A.L., Maher J.M., Augustine L.M., Goedken M.J., Chan J.Y., Cherrington N.J., Klaassen C.D., Manautou J.E. (2008). Induction of Mrp3 and Mrp4 transporters during acetaminophen hepatotoxicity is dependent on Nrf2. Toxicol. Appl. Pharmacol..

[165] Moeller B.J., Cao Y., Li C.Y., Dewhirst M.W. (2004). Radiation activates HIF-1 to regulate vascular radiosensitivity in tumors: Role of reoxygenation, free radicals, and stress granules. Cancer Cell.

[166] Lee S., Lim M.J., Kim M.H., Yu C.H., Yun Y.S., Ahn J., Song J.Y. (2012). An effective strategy for increasing the radiosensitivity of Human lung Cancer cells by blocking Nrf2-dependent antioxidant responses. Free Radic. Biol. Med..

[167] Singh A., Bodas M., Wakabayashi N., Bunz F., Biswal S. (2010). Gain of Nrf2 function in non-small-cell lung cancer cells confers radioresistance. Antioxid. Redox Signal..

[168] Michelson S., Leith J.T. (1994). Dormancy, regression, and recurrence: Towards a unifying theory of tumor growth control. J. Theor. Biol..

[169] Horowitz M., Neeman E., Sharon E., Ben-Eliyahu S. (2015). Exploiting the critical perioperative period to improve long-term cancer outcomes. Nat. Rev. Clin. Oncol..

[170] Tohme S., Yazdani H.O., Al-Khafaji A.B., Chidi A.P., Loughran P., Mowen K., Wang Y., Simmons R.L., Huang H., Tsung A. (2016). Neutrophil Extracellular Traps Promote the Development and Progression of Liver Metastases after Surgical Stress. Cancer Res..

[171] Coffey J.C., Smith M.J., Wang J.H., Bouchier-Hayes D., Cotter T.G., Redmond H.P. (2006). Cancer surgery: Risks and opportunities. Bioessays.

[172] Berguer R., Bravo N., Bowyer M., Egan C., Knolmayer T., Ferrick D. (1999). Major surgery suppresses maximal production of helper T-cell type 1 cytokines without potentiating the release of helper T-cell type 2 cytokines. Arch. Surg..

[173] Antonio N., Bønnelykke-Behrndtz M.L., Ward L.C., Collin J., Christensen I.J., Steiniche T., Schmidt H., Feng Y., Martin P. (2015). The wound inflammatory response exacerbates growth of pre-neoplastic cells and progression to cancer. EMBO J..

[174] Murdoch C., Muthana M., Coffelt S.B., Lewis C.E. (2008). The role of myeloid cells in the promotion of tumour angiogenesis. Nat. Rev. Cancer.

[175] Chang S.H., Liu C.H., Conway R., Han D.K., Nithipatikom K., Trifan O.C., Lane T.F., Hla T. (2004). Role of prostaglandin E2-dependent angiogenic switch in cyclooxygenase 2-induced breast cancer progression. Proc. Natl. Acad. Sci. USA.

[176] Zhang S., Da L., Yang X., Feng D., Yin R., Li M., Zhang Z., Jiang F., Xu L. (2014). Celecoxib potentially inhibits metastasis of lung cancer promoted by surgery in mice, via suppression of the PGE2-modulated β-catenin pathway. Toxicol. Lett..

[177] Wang D., Dubois R.N. (2010). Eicosanoids and cancer. Nat. Rev. Cancer.

[178] Sosnoski D.M., Norgard R.J., Grove C.D., Foster S.J., Mastro A.M. (2015). Dormancy and growth of metastatic breast cancer cells in a bone-like microenvironment. Clin. Exp. Metastasis.

[179] Mao J.T., Smoake J., Park H.K., Lu Q.-Y., Xue B. (2016). Grape Seed Procyanidin Extract Mediates Antineoplastic Effects against Lung Cancer via Modulations of Prostacyclin and 15-HETE Eicosanoid Pathways. Cancer Prev. Res..

[180] Hager P., Permert J., Wikström A.C., Herrington M.K., Ostenson C.G., Strömmer L. (2009). Preoperative glucocorticoid administration attenuates the systemic stress response and hyperglycemia after surgical trauma in the rat. Metabolism.

[181] Desborough J.P. (2000). The stress response to trauma and surgery. Br. J. Anaesth..

[182] Dungan K.M., Braithwaite S.S., Preiser J.-C. (2009). Stress hyperglycaemia. Lancet.

[183] Hou Y., Zhou M., Xie J., Chao P., Feng Q., Wu J. (2017). High glucose levels promote the proliferation of breast cancer cells through GTPases. Breast Cancer.

[184] Li Y., Zhu W., Li J., Liu M., Wei M. (2013). Resveratrol suppresses the STAT3 signaling pathway and inhibits proliferation of high glucose-exposed HepG2 cells partly through SIRT1. Oncol. Rep..

[185] Cao L., Chen X., Xiao X., Ma Q., Li W. (2016). Resveratrol inhibits hyperglycemia-driven ROS-induced invasion and migration of pancreatic cancer cells via suppression of the ERK and p38 MAPK signaling pathways. Int. J. Oncol..

[186] Garufi A., D’Orazi G. (2014). High glucose dephosphorylates serine 46 and inhibits p53 apoptotic activity. J. Exp. Clin. Cancer Res..

[187] Biernacka K.M., Uzoh C.C., Zeng L., Persad R.A., Bahl A., Gillatt D., Perks C.M., Holly J.M. (2013). Hyperglycaemia-induced chemoresistance of prostate cancer cells due to IGFBP2. Endocr. Relat. Cancer.

[188] Sato K., Hikita H., Myojin Y., Fukumoto K., Murai K., Sakane S., Tamura T., Yamai T., Nozaki Y., Yoshioka T. (2020). Hyperglycemia enhances pancreatic cancer progression accompanied by elevations in phosphorylated STAT3 and MYC levels. PLoS ONE.

[189] Webster K.A. (2008). Stress hyperglycemia and enhanced sensitivity to myocardial infarction. Curr. Hypertens. Rep..

[190] Giri B., Dey S., Das T., Sarkar M., Banerjee J., Dash S.K. (2018). Chronic hyperglycemia mediated physiological alteration and metabolic distortion leads to organ dysfunction, infection, cancer progression and other pathophysiological consequences: An update on glucose toxicity. Biomed. Pharmacother..

[191] Xiao Y., Yu D. (2021). Tumor microenvironment as a therapeutic target in cancer. Pharmacol. Ther..

[192] Miret Durazo C.I., Zachariah Saji S., Rawat A., Motiño Villanueva A.L., Bhandari A., Nurjanah T., Ryali N., Zepeda Martínez I.G., Cruz Santiago J.A. (2024). Exploring Aspirin’s Potential in Cancer Prevention: A Comprehensive Review of the Current Evidence. Cureus.

[193] Guo C.-G., Ma W., Drew D.A., Cao Y., Nguyen L.H., Joshi A.D., Ng K., Ogino S., Meyerhardt J.A., Song M. (2021). Aspirin Use and Risk of Colorectal Cancer Among Older Adults. JAMA Oncol..

[194] Elwood P., Morgan G., Watkins J., Protty M., Mason M., Adams R., Dolwani S., Pickering J., Delon C., Longley M. (2024). Aspirin and cancer treatment: Systematic reviews and meta-analyses of evidence: For and against. Br. J. Cancer.

[195] Baker A., Kartsonaki C. (2023). Aspirin Use and Survival Among Patients With Breast Cancer: A Systematic Review and Meta-Analysis. Oncologist.

[196] Chen W.Y., Ballman K.V., Partridge A.H., Hahn O.M., Briccetti F.M., Irvin W.J., Symington B., Visvanathan K., Pohlmann P.R., Openshaw T.H. (2024). Aspirin vs Placebo as Adjuvant Therapy for Breast Cancer: The Alliance A011502 Randomized Trial. JAMA.

[197] Drew D.A., Chan A.T. (2021). Aspirin in the Prevention of Colorectal Neoplasia. Annu. Rev. Med..

[198] Hou J., Karin M., Sun B. (2021). Targeting cancer-promoting inflammation—Have anti-inflammatory therapies come of age?. Nat. Rev. Clin. Oncol..

[199] Brown M., Cohen J., Arun P., Chen Z., Van Waes C. (2008). NF-kappaB in carcinoma therapy and prevention. Expert. Opin. Ther. Targets.

[200] Sordo-Bahamonde C., Lorenzo-Herrero S., Gonzalez-Rodriguez A.P., Martínez-Pérez A., Rodrigo J.P., García-Pedrero J.M., Gonzalez S. (2023). Chemo-Immunotherapy: A New Trend in Cancer Treatment. Cancers.

[201] Amouzegar A., Chelvanambi M., Filderman J.N., Storkus W.J., Luke J.J. (2021). STING Agonists as Cancer Therapeutics. Cancers.

[202] Barber G.N. (2015). STING: Infection, inflammation and cancer. Nat. Rev. Immunol..

[203] Zhu Y., An X., Zhang X., Qiao Y., Zheng T., Li X. (2019). STING: A master regulator in the cancer-immunity cycle. Mol. Cancer.

[204] Fu J., Kanne D.B., Leong M., Glickman L.H., McWhirter S.M., Lemmens E., Mechette K., Leong J.J., Lauer P., Liu W. (2015). STING agonist formulated cancer vaccines can cure established tumors resistant to PD-1 blockade. Sci. Transl. Med..

